# NEMUCO: The In Vitro 4D NMJ as an Innovative Powerful Tool to Study Nerve and Muscle Cell–Cell Communication

**DOI:** 10.3390/ijms27208946

**Published:** 2026-10-09

**Authors:** Katharina Block, Gaia Ziraldo, Gabor Trautmann, Sandra Furlan, Abhishek Singh, Sabine Grosser, Martina Gutsmann, Paola Lorenzon, Pompeo Volpe, Imre Vida, Dieter Blottner, Michele Salanova

**Affiliations:** 1 Institute of Integrative Neuroanatomy, Charité—Universitätsmedizin Berlin, 10115 Berlin, Germany; kablock.email@gmail.com (K.B.); gaia.ziraldo@charite.de (G.Z.); gabor.trautmann@charite.de (G.T.); abhisheksinghbandral2@gmail.com (A.S.); sabine.grosser@charite.de (S.G.); martina.gutsmann@charite.de (M.G.); imre.vida@charite.de (I.V.); dieter.blottner@charite.de (D.B.); 2 Section of Padova, CNR-Neuroscience Institute, 35131 Padova, Italy; sfurlan@bio.unipd.it; 3 Department of Life Sciences, University of Trieste, 34127 Trieste, Italy; plorenzon@units.it; 4 Department of Biomedical Sciences, University of Padova, 35121 Padova, Italy; pompeo.volpe@unipd.it

**Keywords:** muscle and nerve cell co-culture, muscle synaptogenesis in vitro, neuromuscular junction development

## Abstract

Chronic muscle unloading, following denervation, aging, disease, or long-term bed rest, whether as a spaceflight analog or during an actual spaceflight mission, results in a decline in structure and function of the neuromuscular junction (NMJ). This decline leads to a decrease in muscle mass and impaired fine motor control. Similar changes are also observed in several neuromuscular diseases and myopathies’ impaired movement control symptoms. Here, we report on a powerful 3D co-culture experimental model developing NMJ-like structures, as a novel in vitro platform for functional/regulatory studies during time (4D co-culture) for basic and translational research purposes. Murine NErve (NSC-34 motoneuron) and MUscle (C2C12 myoblast) cell CO-cultures (NEMUCO) were grown on either synthetic or biological three-dimensional (3D) scaffolds. Morphological analysis obtained through 3D reconstruction in proximity of the cell–cell contact revealed close apposition between NSC-34 motoneurons and C2C12 myotubes. The co-cultures were further analyzed using a significant array of molecular and cellular biology tools, based on NMJ-specific molecular marker expression combined with muscle- and nerve-cell-specific differentiation biomarkers. Specialized cell–cell contacts were present starting at day 2 of co-culture. Triple immunostaining indicated neurofilament-positive axonal nerve terminals approaching clustered α-bungarotoxin-positive nicotinic acetylcholine receptors (nAChRs) in desmin- or fast-type myosin heavy chain (fMyHC)-positive developing myotubes, representing the first signs of NMJ-like structure assembly in our in vitro system. It is noteworthy that co-cultured motoneurons showed a trend of increase in SNAP-25 transcription, one of the most important SNARE proteins, whereas co-cultured myotubes showed a slight increase in junctophilin and ryanodine receptor type 1 (RyR1) transcription, both critical proteins of the triadic junction of differentiated muscle fibers. In most respects, neuronal-myotube cell–cell contacts in our 3D co-cultures mimicked native pre- and postsynaptic NMJ microdomains. Neuronal-myotube co-cultures grown in 3D scaffolds represent a powerful tool for investigating the molecular mechanisms underlying NMJ adaptation and plasticity in muscle myopathies. Moreover, this approach could be adapted for customized miniaturized platforms designed for cellular neurobiology research under spaceflight conditions.

## 1. Introduction

Chronic muscle unloading during disuse, disease, aging and/or spaceflight missions results in a significant decline in neuromuscular junction (NMJ) structure and function, which in turn has a significant impact on muscle mass maintenance and impairs fine motor control [1,2,3,4,5,6,7,8]. Therefore, a deeper understanding of the molecular and cellular mechanisms that lead to NMJ imbalance is of paramount importance in order to establish reliable countermeasure approaches.

The NMJ is a unique specialized muscle and nerve cell–cell communication contact that translates neuronal electrical impulses into skeletal muscle mechanical responses [9,10,11]. The fusion of presynaptic vesicles containing the neurotransmitter acetylcholine (ACh) at the nerve terminal membrane provides a unique and highly efficient mechanism responsible for the selective activation of postsynaptic nicotinic ACh receptors (nAChRs) on the postsynaptic skeletal muscle membrane [12], which is required for muscle contraction [9,10].

Moreover, skeletal muscle not only contracts during movement but also fulfills many other vital physiological functions such as muscle tone, tissue homeostasis and different intra- and extra-synaptic myonuclear gene expression and translational regulation as well as synaptic plasticity [13]. Therefore, the mechanisms that translate neuronal electrical stimuli into skeletal muscle transcriptional programs and mechanical responses at the NMJ are very complex and tightly regulated, requiring a high degree of coordination between different signaling pathways and their eventual cross-talk in normal muscle function.

To date, the underlying molecular mechanisms and signaling pathways that negatively impact NMJ performance in different physiological and pathophysiological conditions remain largely unexplored.

Due to the unique characteristics of the NMJ and its complexity, in addition to technical and ethical limitations, it remains difficult to study it in human muscle in vivo, especially when it comes to pharmacological treatment.

With the aim of further investigating such mechanisms many efforts have been made in the last decade to establish a controlled experimental model to generate the NMJ in vitro as close as possible to the native form. In this regard, primary cell co-cultures were initially used [14]. However, given the need for standardized experimental models, the use of immortalized cell lines, for both muscle and motoneuron cells, was adopted. Only recently, however, following the development of the innovative three-dimensional (3D) co-culture methodology and the growing interest in in vitro cell biology, induced pluripotent stem cells such as iPSC cells have been employed [15,16]. The use of iPSC cells allows multiple cell types and tissues to be organized within complex in vitro systems, which are defined as organoids, ultimately making them far more complex than 3D in vitro systems [17].

Overall, the use of 3D co-cultures offers a more realistic model of the physiological environment. It allows for more complex interactions between different cell types and a better imitation of tissue functionality, with greater robustness and longevity of the cell culture itself. Furthermore, this provides an interesting platform for evaluating the behavior of new chemical entities in drug development, leading to improved applications in the development of drug therapies. No less important, the application of such an experimental model could be of particular interest and a useful tool to better investigate and further elucidate the molecular mechanisms underlying the cell–cell communication contacts between muscle cells and nerve cells in different experimental environmental conditions. This experimental model could represent a powerful tool to explore the spatial and temporal hierarchical assembly of the different molecular players at the pre- and postsynaptic sides, their associated signaling pathways and their potential cross-talk during muscle synaptogenesis, avoiding the use of animals. Indeed, this experimental model can be successfully implemented in engineered hardware for space science research experiments in order to further investigate molecular mechanisms of disuse-induced skeletal muscle atrophy in astronauts during long-duration missions. Notably, highly preserved cell viability was recently observed in NEMUCO co-cultures integrated into cellBox-3 (ScienceShells) hardware for 12 days on the ground (Salanova’s lab communication).

We established a novel experimental protocol for an advanced 4D co-culture system in which differentiated nerve and muscle cells were grown on 3D scaffolds. These cells were able to interact with each other over time (as the 4th dimension), generating NMJ-like structures in vitro. Furthermore, our data demonstrated that our experimental model is characterized by an advanced degree of cell maturation and up-regulation of the myogenic program inherent to normal muscle development. This protocol can be adapted to investigate several models of NMJ-associated pathologies, and could provide deeper insight into fundamental aspects of NMJ development, maturation and maintenance that are still not well understood.

## 2. Results

### 2.1. Native NMJ of the Rat Soleus Skeletal Muscle

**Figure 1:**
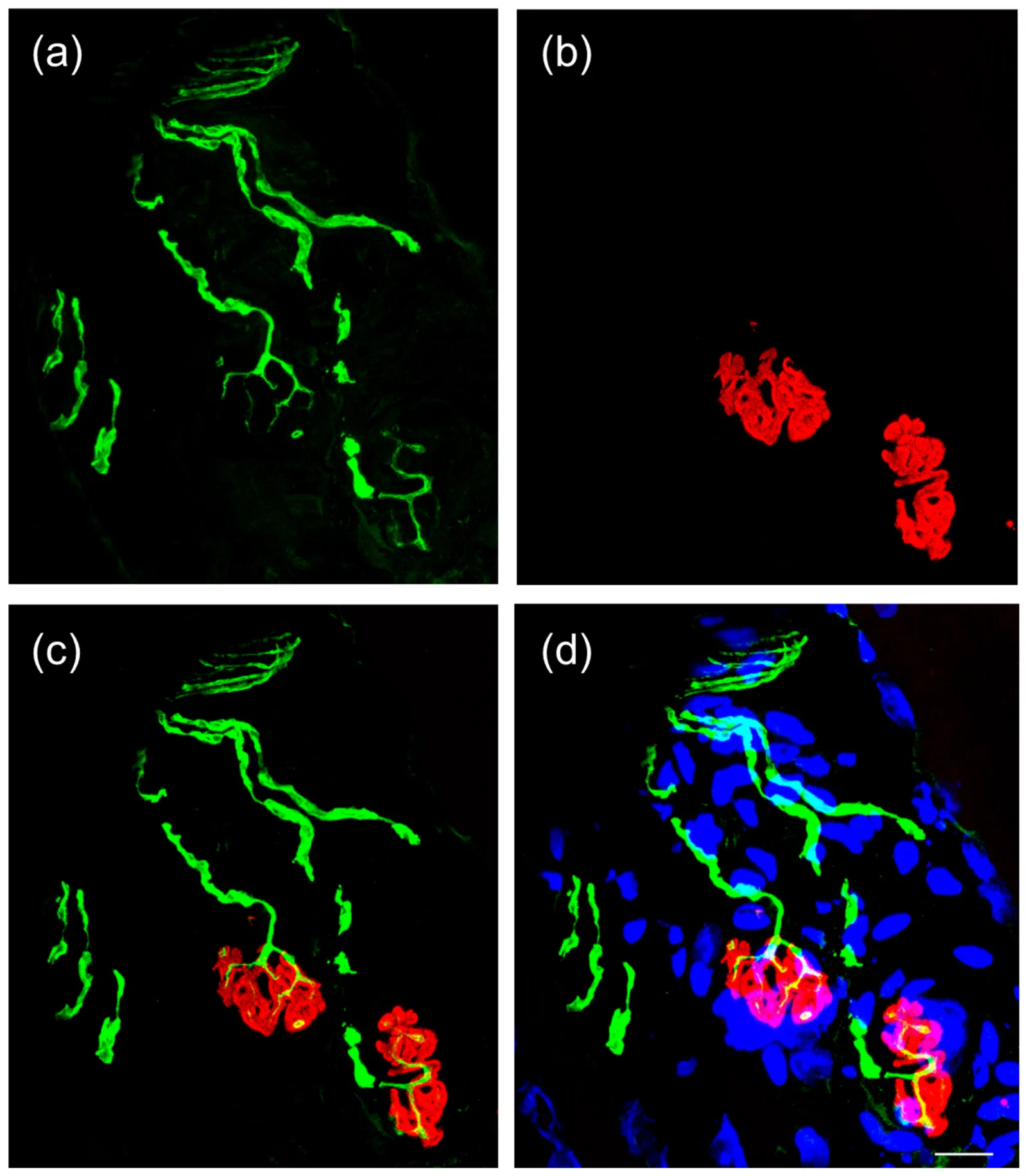
Laser confocal microscopy 3D reconstruction of the native NMJ of the skeletal muscle soleus of the rat. (**a**) Representative image of native NMJ stained with anti-neurofilament antibodies (green); (**b**) α-bungarotoxin (α-BTX, red), a specific marker of nAChRs; (**c**) merged image with anti-neurofilament antibodies (green) and α-BTX (red); (**d**) merged image also counterstained nuclei/DAPI (blue). Maximal projection of 10 stacks, 1 µm each. Magnification bar = 10 µm.

Native NMJs were stained in the soleus muscle of rodents according to [1]. Several NMJs were identified and used as native reference controls. Figure 1 shows a typical NMJ from the soleus muscle without myonuclear staining (Figure 1c) and with subsynaptic and extra-synaptic myonuclear staining (Figure 1d).

### 2.2. In Vitro Development of NMJs Using Non-Canonical (Synthetic) and Canonical (Biological) 3D Scaffold

To develop a reliable 3D experimental model, we established a 3D co-culture protocol for nerve and muscle cells capable of forming NMJ-like structures in vitro.

For this purpose, the mouse C2C12 myoblast cell line was used as a muscle model [18], whereas the mouse NSC-34 motoneuron cell line served as the neuronal counterpart [19].

Two different 3D scaffolds were tested: a synthetic Alvetex^®^ polystyrene scaffold [20] and a modified biological scaffold consisting of highly purified extracellular matrix (ECM) hydrogel [21]. In both types of 3D scaffolds, NMJs were found at various developmental stages.

Ten co-cultures were set up for each type of scaffold. Each culture consisted of six to ten samples, with cells cultured on either 1 cm^2^ Alvetex scaffolds for the synthetic scaffold or round glass slide coverslips with an 18 mm diameter for the biological/hydrogel scaffold. We observed 5 to 9 nerve and muscle cell–cell contacts per sample for each of the two types of scaffolds examined.

### 2.3. Nerve and Muscle Co-Cultures on a Synthetic/Alvetex 3D Scaffold

The Alvetex^®^ polystyrene scaffold consists of a highly porous cross-linked polystyrene material that is 200 µm thick and contains communicating voids with an average diameter of 42 µm. The material is designed for three-dimensional culture of mammalian cells within the scaffold. At optimal cell density, cells coexist in the same micro-niche of this highly networked material, increasing the likelihood of cell interactions and, in the case of muscle and nerve cell co-cultures, facilitating the development of specialized cell–cell contacts such as NMJs.

**Figure 2:**
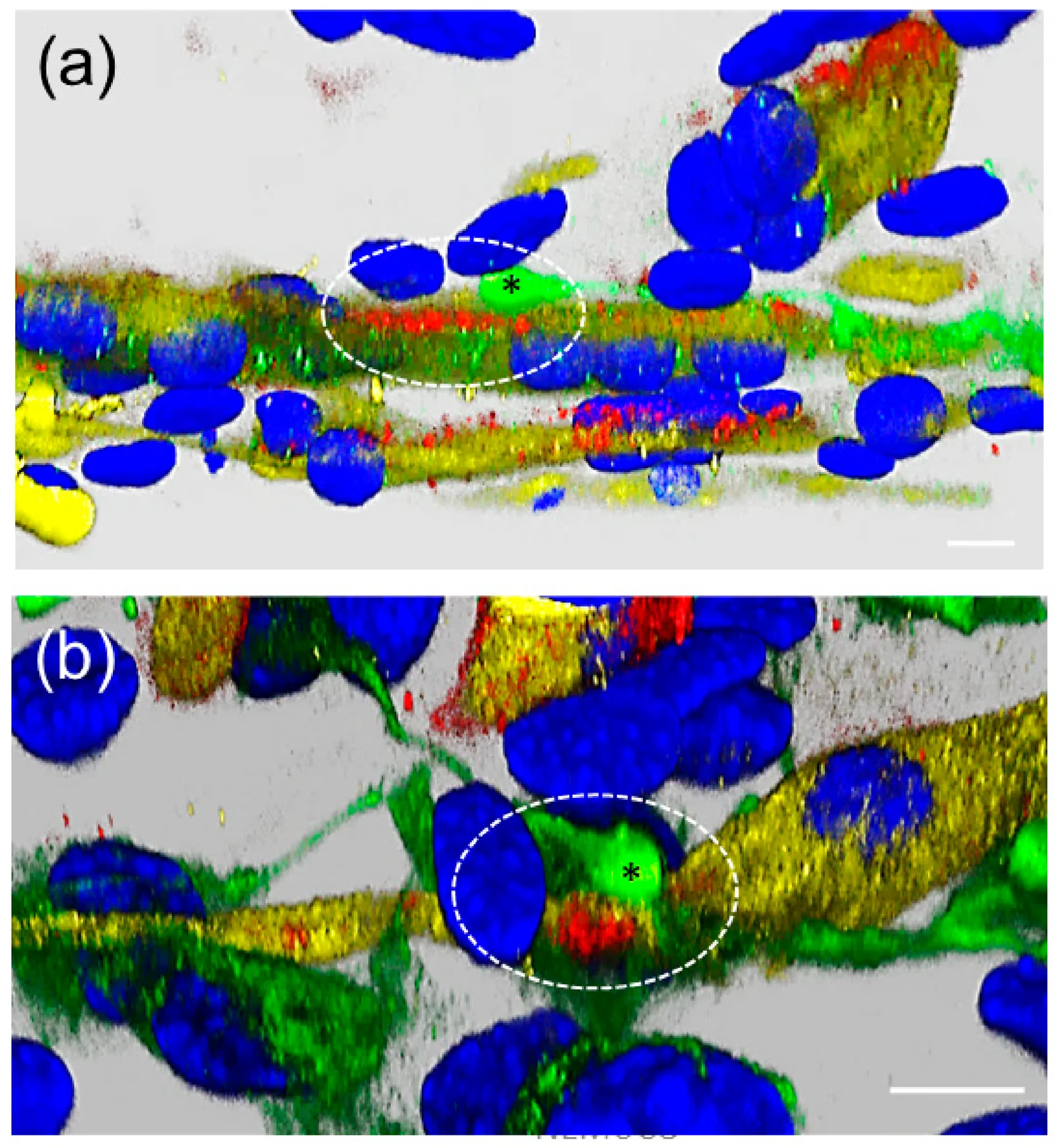
Laser confocal microscopy 3D reconstruction of nerve and muscle cells 3D co-cultured on a synthetic Alvetex polystyrene scaffold. (**a**) Representative image from early, day 2; and (**b**) late, day 5, stage of 3D co-cultures immunolabelled for neurofilaments (green) and desmin (yellow). nAChRs were stained with α-BTX (red) and nuclei counterstained with DAPI (blue). White dashed oval circles: area of nerve and muscle cell–cell contact. Note that the nerve endings (asterisks) approach the muscle cells at the level of prepatterned nAChR clusters. Magnification bar = 12 µm.

C2C12 myoblasts and NSC-34 motoneurons were seeded on scaffolds precoated with laminin and collagen I, cultured for up to 12–14 days in medium containing nutrients and muscle- and nerve-specific growth factors (for details see Methods), and processed for immunocytochemistry at 3, 7, and 12 days of culture. Figure 2 shows a 3D reconstruction of a typical immunocytochemical image. Desmin was used as an early muscle differentiation marker; this protein forms a subsarcolemmal network across the myotube. Neurofilament proteins were used to label motoneuron axons, and α-BTX was used to label nAChRs.

As shown in Figure 2a,b (dashed circles), myotubes co-cultured with motoneurons on the 3D synthetic scaffold developed cell–cell contacts. Axons stained with anti-neurofilament antibodies terminated above the myotube near prepatterned nAChRs, suggesting developing communication contacts between myotubes and neuronal cells.

Fluorescence staining with α-BTX revealed progressive, time-dependent aggregation of nAChRs in the myotube plasma membrane, particularly near cell–cell contacts and nerve endings, suggesting the formation of NMJ-like structures in our 3D co-cultures. Progressive nAChR clustering was evident between days 2 and 5 of co-culture (Figure 2a,b, dashed circles).

By means of 3D morphological reconstruction analysis, we observed only a few myonuclei in each myotube, suggesting limited myoblast fusion during early myotube development on the 3D synthetic scaffold, possibly due to spatial constraints within the highly porous polystyrene network.

The 3D co-culture system on a polystyrene synthetic scaffold very likely produced NMJ-like structures in a proper, regular, developing manner, showing muscle-specific differentiation markers such as contractile filaments.

However, the opaque nature of the polystyrene scaffold and the deep positioning of cells concealed within its porous structure precluded real-time phase contrast and differential interference contrast (DIC) microscopy for proper visualization.

### 2.4. Nerve and Muscle Cell Co-Cultures on Biological 3D Scaffolds

To circumvent the problems of low myonuclear number and small myotube size, we encapsulated muscle and nerve cells in extracellular matrix hydrogels, including Matrigel and collagen I (see Section 4). The advantages of using such hydrogels include (i) ease of processing, (ii) low cost, and (iii) flexibility in live cell manipulation. In addition, pore size, ligand density, and hydrogel stiffness can be adjusted by modifying the concentration of matrix components, thereby facilitating modification of the hydrogel’s microstructural properties. Thus, the hydrogel supports cell differentiation by providing an environment that mimics the native extracellular matrix (ECM) in vivo. NSC-34 motoneurons were seeded on top of the hydrogel containing C2C12 myotubes, creating spatially distinct microenvironments, while supporting cell–cell interactions and subcellular compartments.

Three different hydrogel concentrations were initially tested to identify the optimal formulation supporting myoblast differentiation and fusion, as well as motoneuron differentiation and axonal sprouting. The optimal formulation consisted of 36% collagen I and 14% Matrigel, which provided ideal conditions for both muscle and nerve cell differentiation.

**Figure 3:**
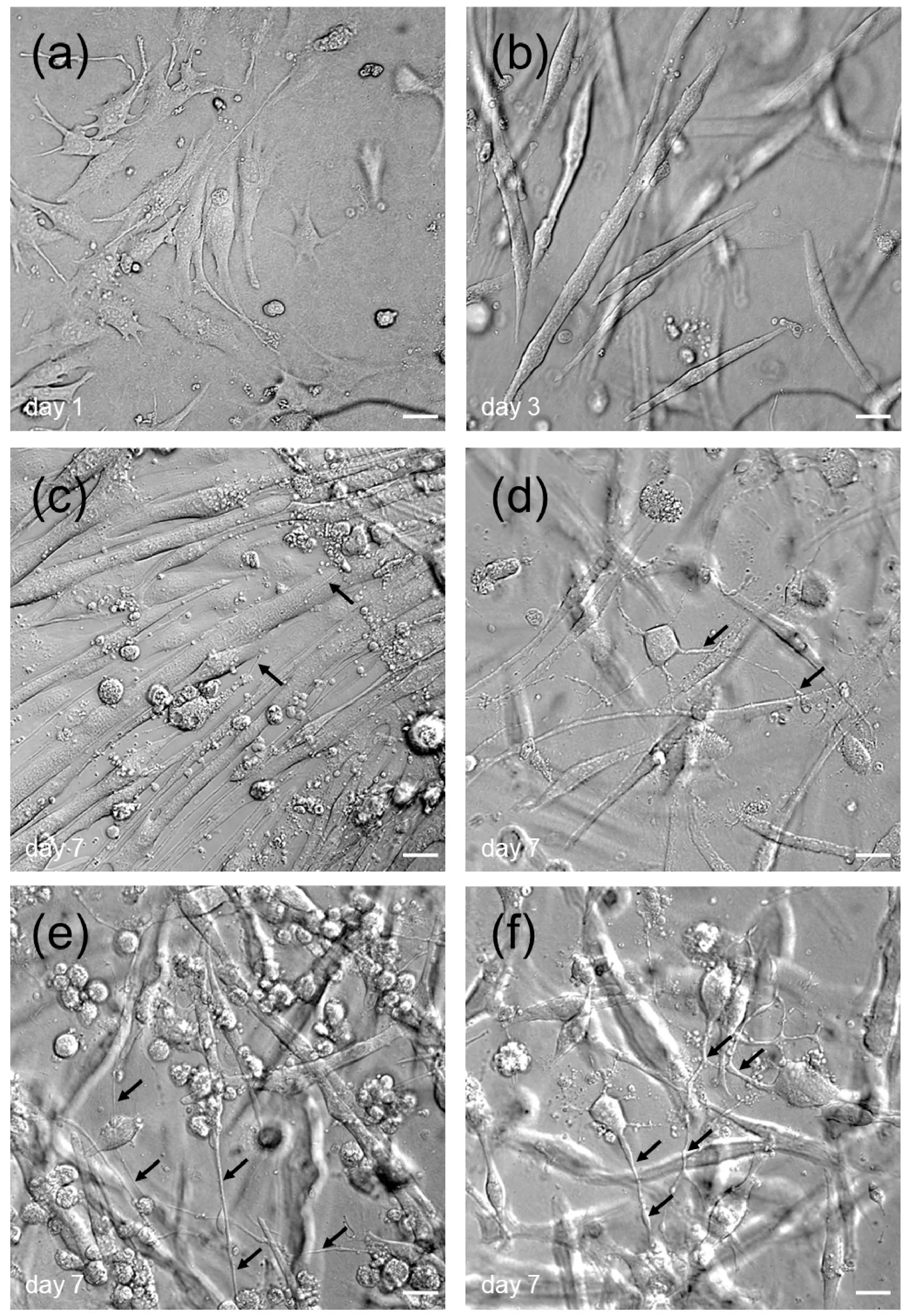
Differential interference contrast (DIC) microscopy of differentiating mouse C2C12 myoblasts and NSC-34 motoneurons in 3D co-cultures. (**a**) Proliferating C2C12 myoblasts, 1-day in vitro culture. (**b**) Differentiating and fusing C2C12 myoblasts, 3 days in culture. (**c**) Maturing C2C12 myotubes with a typical cross-striated pattern and an NSC-34 motoneuron with a prominent axon (arrows) are clearly visible, 7 days in co-culture. (**d**) NSC-34 motoneuron (top) with a prominent axon (arrows) and myotubes (bottom) are visible, 7 days in co-culture. (**e**,**f**) Co-cultures with several C2C12 myotubes and NSC-34 motoneurons with a prominent axon (arrows) at different *z*-axis levels. Magnification bar: 25 µm.

Figure 3a–f show different developmental stages of the 3D muscle and nerve cell co-culture as visualized by DIC. Figure 3a shows proliferating C2C12 myoblasts at culture day 1. Figure 3b shows elongated myoblasts fusing into myotubes. Figure 3c shows maturing myotubes with a typical cross-striated pattern, indicating increased assembly of the contractile apparatus. Figure 3d–f show individual NSC-34 motoneurons (arrows) contacting myotubes.

Notably, myotubes cultured in the hydrogel scaffold contained an elevated number of myonuclei compared to those grown on a polystyrene scaffold, indicating enhanced myoblast fusion in the latter case. Co-cultures were maintained for 1–2 weeks to allow complete differentiation.

**Figure 4:**
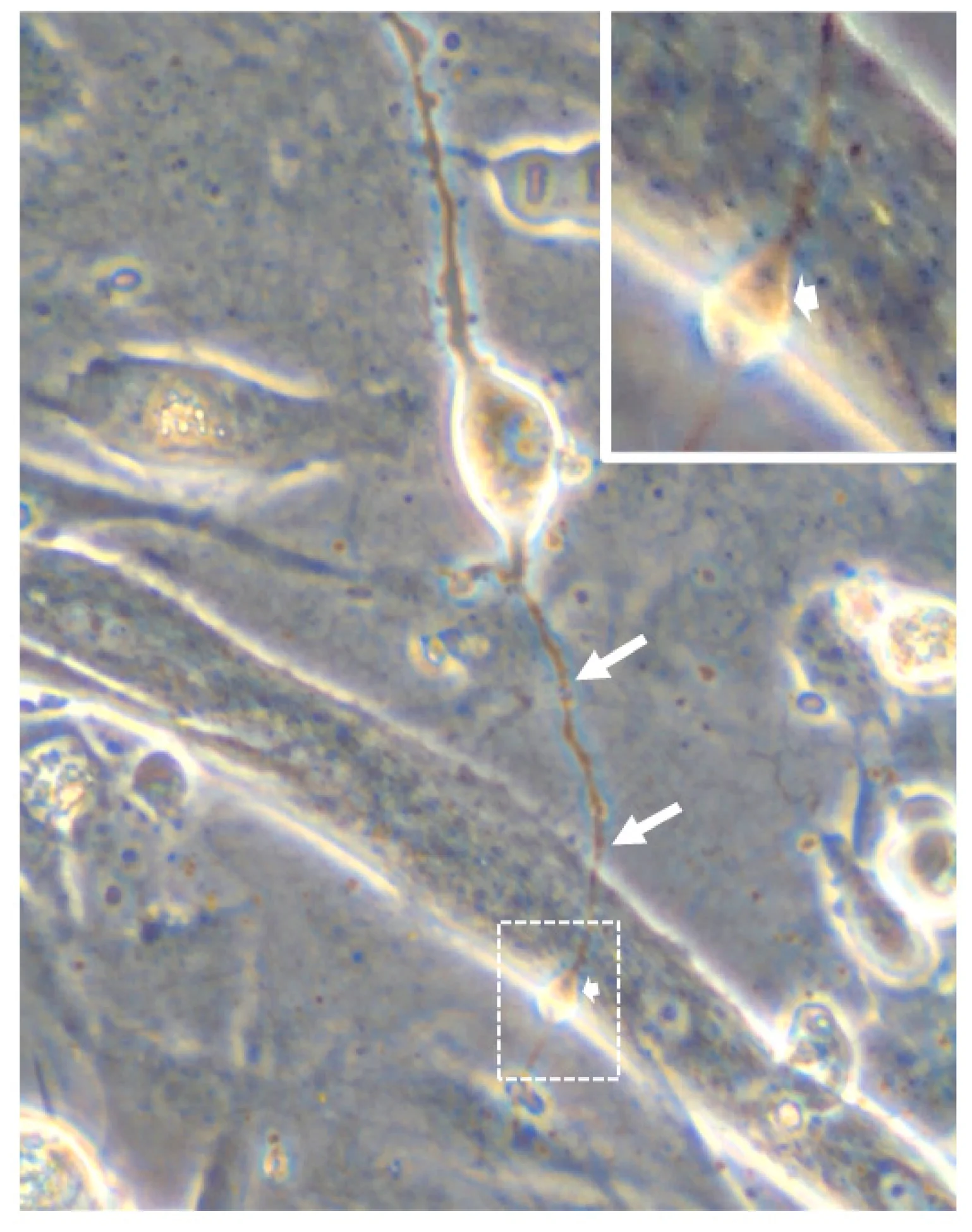
Bright-field image of mouse C2C12 muscle myotubes and NSC-34 motoneuron 3D co-culture on a hydrogel scaffold. A well-differentiated NSC-34 motoneuron with a relatively long axon (arrows) is present in contact with a small C2C12 myotube and, more likely, the large myotube located at a lower level (5 days of co-culture). White dashed box: developing specialized muscle and nerve cell–cell contact (arrowhead) close to the nerve ending. Inset: Magnification of the dashed box showing the nerve ending (arrowhead) in contact with the myotube and a branched axon extending above a differentiated myotube. A representative frame was extracted from a live-cell video acquired with a Leica microscope (Obj. 40x) using the HD camera MC170.

Figure 4 shows a bright-field image from a live-cell video of the co-culture. Arrows indicate a motoneuron axon extending toward a myotube. The arrowhead (inset TOP right) marks the nerve terminal in contact with the myotube (dashed box in overview), suggesting a developing NMJ-like structure. The inset shows a magnified view of the contact site (arrowhead).

To assess muscle cell maturation and the formation of NMJ-like structures in the hydrogel scaffold, we performed immunocytochemistry experiments using muscular differentiation markers and NMJ-specific markers.

**Figure 5:**
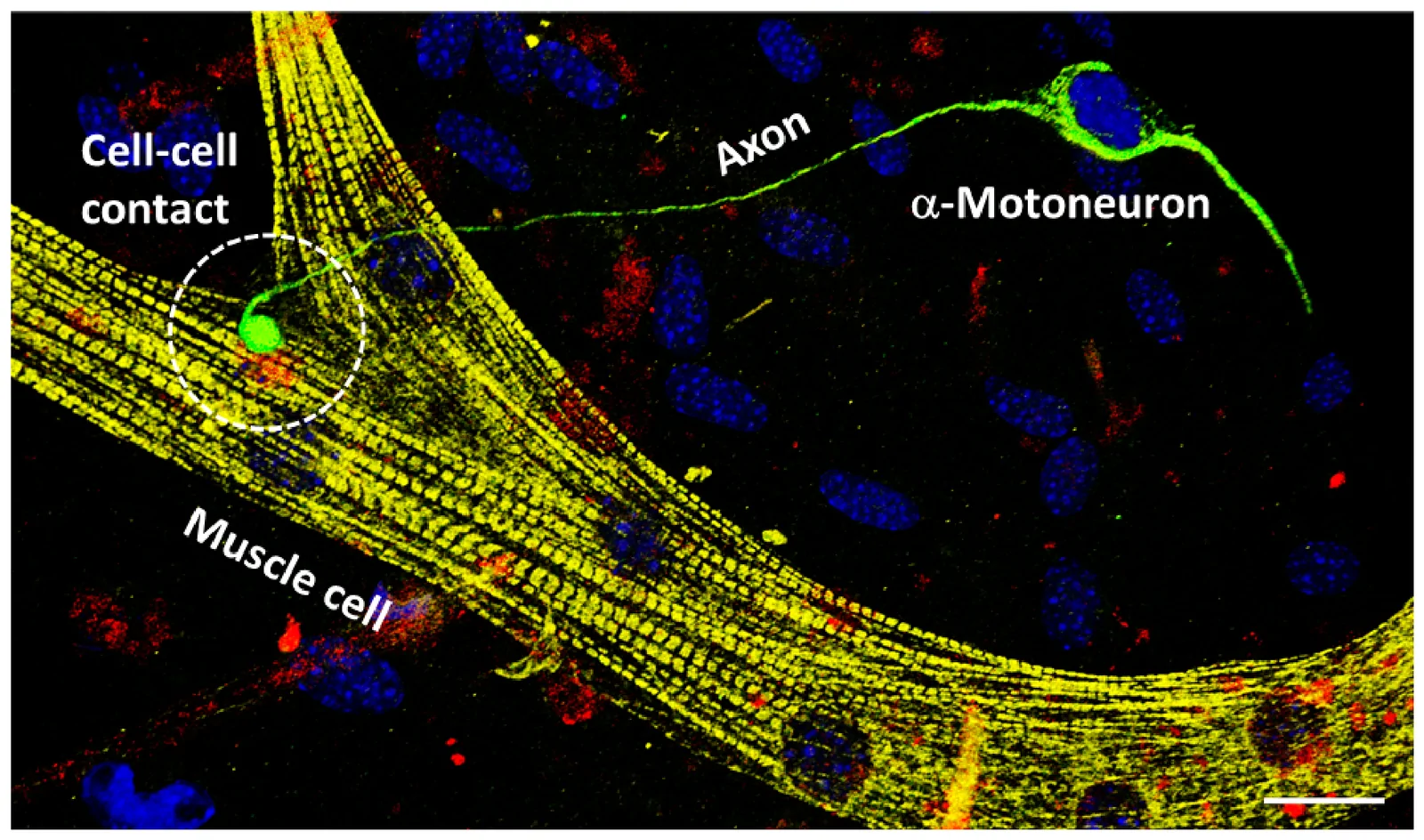
Laser confocal microscopy of nerve and muscle 3D co-culture. Triple-staining experiment in C2C12 myotubes and NSC-34 motoneurons for fMyHC (yellow), neurofilament (green) and nAChRs (red) (7 days in co-culture). Nuclei were counterstained with DAPI (blue). White dashed ring: axon terminal ending contact on pre-patterned nAChRs, suggesting the presence of developing NMJ-like structures in the co-culture. Maximal projection of 10 stacks, 1 µm each. Magnification bar = 15 µm.

**Figure 6:**
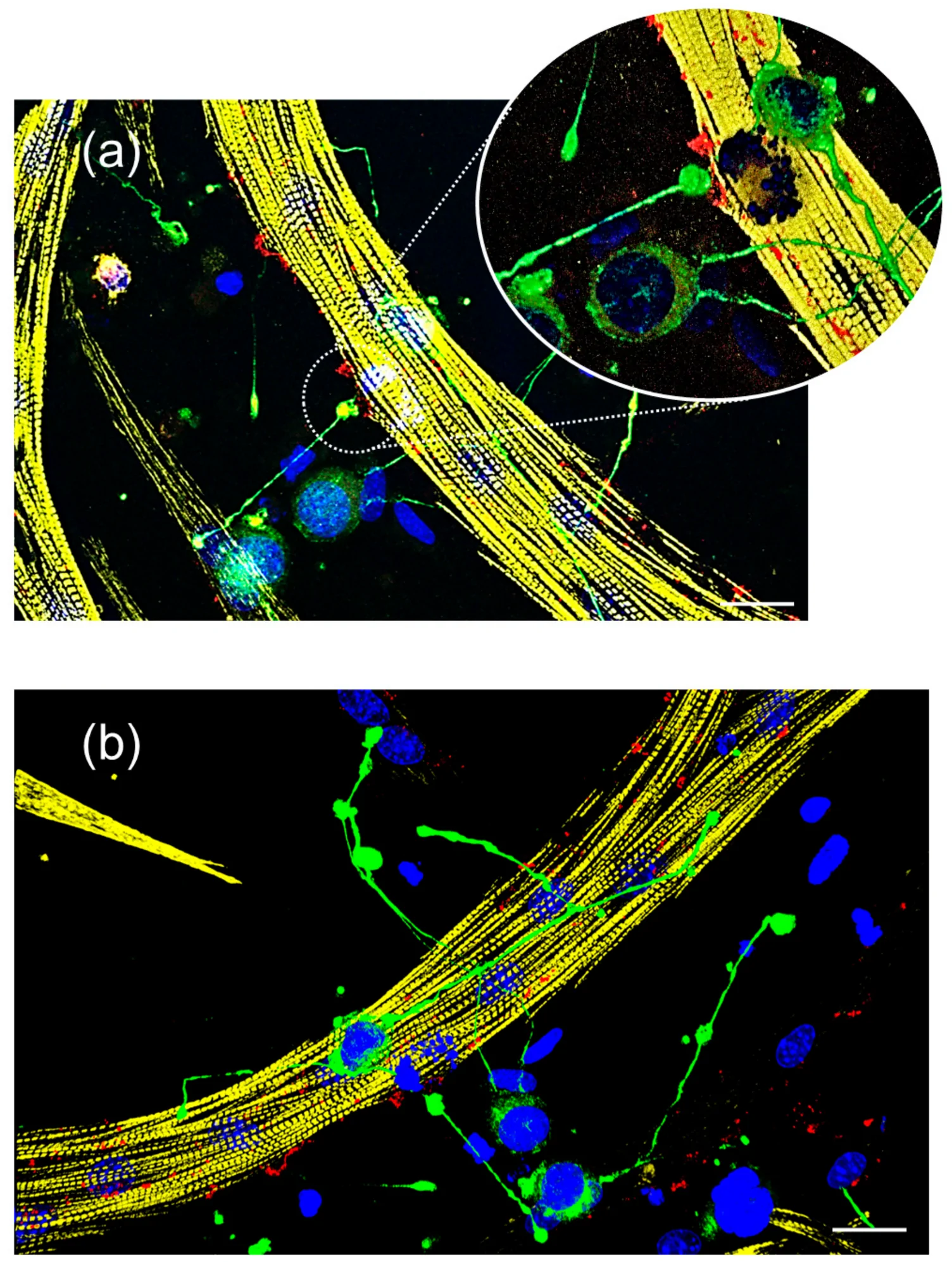
Laser confocal microscopy 3D reconstruction of muscle and nerve cell co-cultures in a hydrogel scaffold within the CellBox hardware. (**a**) C2C12 maturing myotubes stained for fMyHC (yellow), neurofilaments (green) and prepatterned nAChRs (red); nuclei were counterstained with DAPI (blue). The insert shows a flipped image, with the connection between nerve and muscle cell–cell contact in the foreground. (**b**) Co-culture immunostaining as in (**a**), showing a different area of the same field with a higher density of motoneurons (NSC-34) and longer axons (green). Maximal projection of 10 stacks, each 1 µm. Magnification bar = 15 µm.

Figure 5 and Figure 6 show the expression pattern of fast-type myosin heavy chain (fMyHC) in co-cultured myotubes. Notably, fMyHC immunohistochemistry experiments revealed that myotubes contacted by α-motoneurons exhibited improved organization of the cytoskeleton and contractile apparatus, forming cross-striated patterns characteristic of sarcomere-like structures in adult skeletal muscle (Figure 5 and Figure 6).

To further study NMJ-like structure development and stability under microgravity experimental culture conditions, cells were also grown in the CellBox-3 (ScienceShells) hardware on the ground, i.e., a closed, automated cell life support system that allows programmed medium exchange and the use of fixative reagent at the end of the experiment. This served as a pilot experiment for the planned NEMUCO spaceflight mission aboard the International Space Station (ISS).

Figure 6 shows that NEMUCO co-cultures thrived in the perfused microchambers. Notably, the co-cultures formed cell–cell contacts also within the CellBox-3 hardware, validating the system’s suitability for spaceflight experiments.

**Figure 7:**
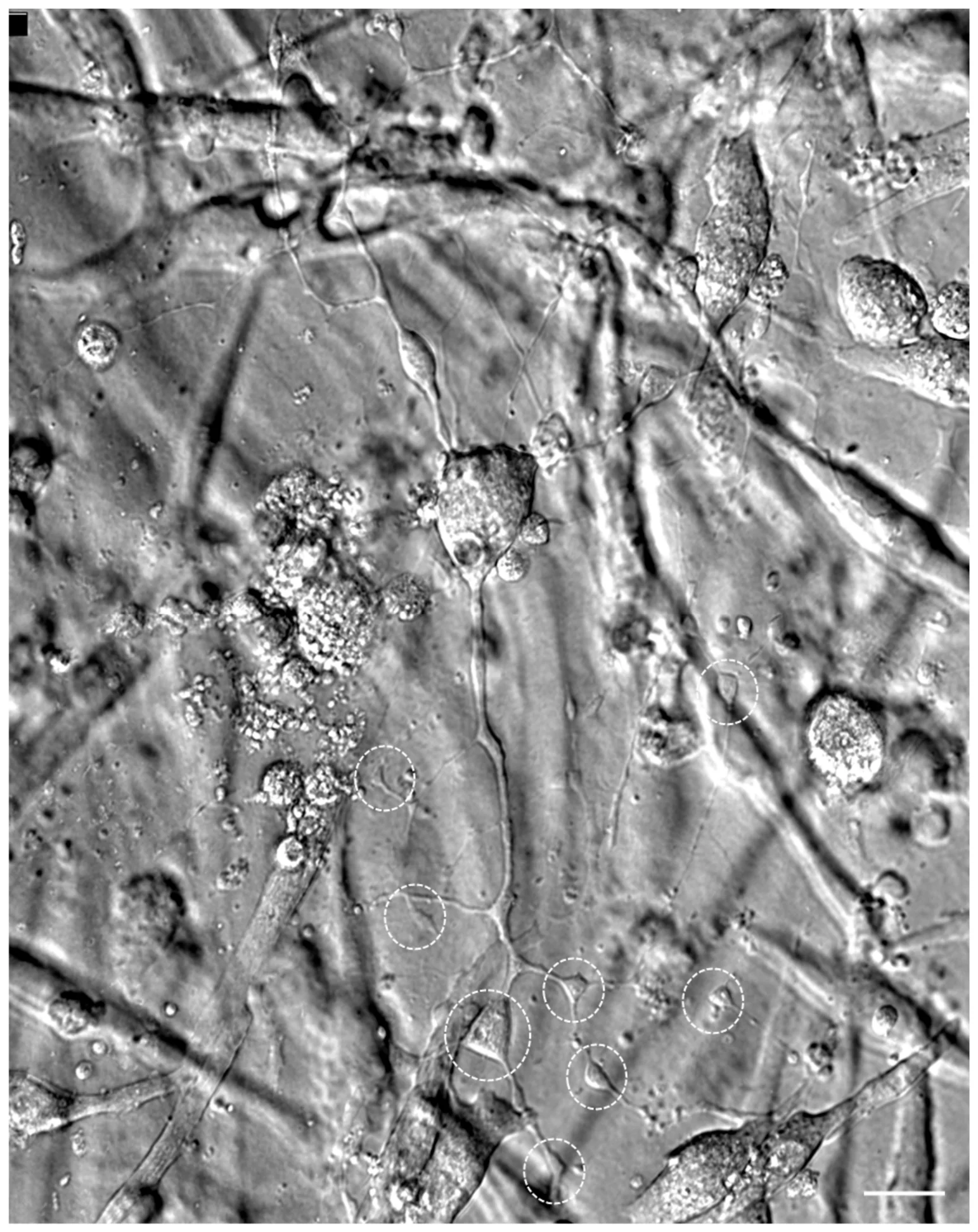
Differential interference contrast (DIC) microscopy of motor-unit-like network structures formed by differentiating mouse C2C12 myoblasts and NSC-34 motoneurons in 3D co-culture on a hydrogel scaffold. NSC-34 motoneuron and C2C12 myotube cells in focus. NSC-34 motoneuron at the top, with a prominent branched axon and nerve terminals (dashed circle), and several C2C12 myotubes at the bottom are visible (7 days in co-culture). Magnification bar = 25 µm.

DIC provides a reliable, high-resolution tool to investigate 3D structures of cell co-cultures and, in particular, unambiguous visualization of specialized cell–cell contacts. DIC microscopic imaging was performed on the 3D co-cultures grown in hydrogel. Figure 7 shows a representative DIC image revealing a motor unit-like networking structure in vitro (dashed rings). As shown in Figure 7, a large NSC-34 motoneuron positioned above the C2C12 myotubes with its long-branched axon and nerve terminals contacts several myotubes located at different focal planes (dashed circles), suggestive of typical NMJ-like structures and thus recapitulating the classic feature of a motor unit.

Taken together, these results demonstrate that co-cultures of NSC-34 motoneuron and C2C12 muscle cell lines grown on biological hydrogel 3D scaffolds develop specialized nerve-muscle cell–cell contacts, as evidenced by the expression of nerve-, muscle-, and NMJ-specific molecular markers.

### 2.5. Nerve-Contact Independent nAChRs Clustering on Biomatrix 3D Scaffolds

**Figure 8:**
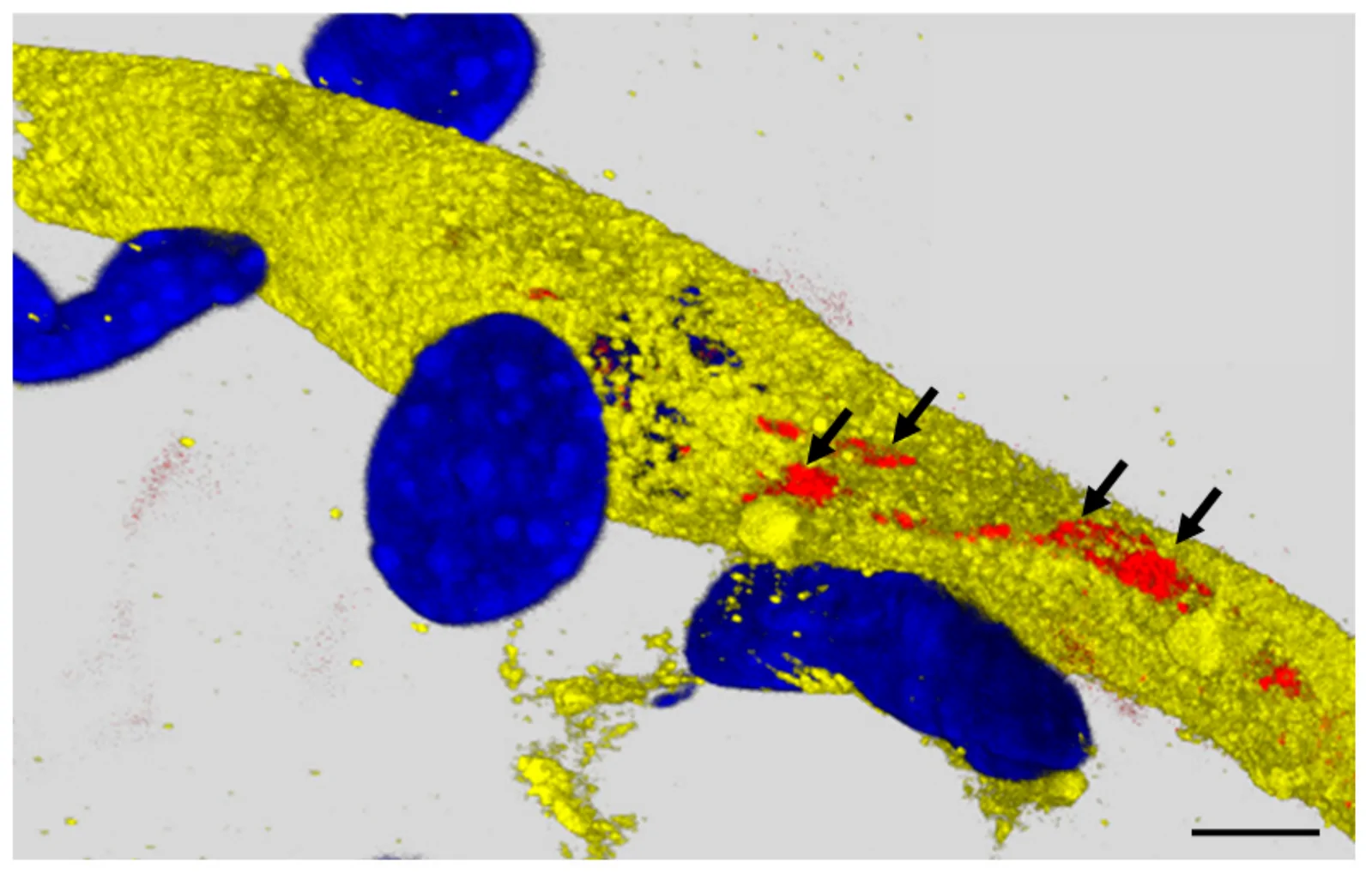
Laser confocal microscopy 3D reconstruction of clustered nAChRs in cultured immature myotubes in the absence of NSC-34 motoneuron direct contact. C2C12 myotubes were triple stained for desmin (yellow), nAChRs (red) and nuclei (blue). Arrows indicate patterned nAChRs. Maximum projection of 10 stacks, each 1 µm. Magnification bar = 15 µm.

Figure 8 shows the patterning of nAChRs in muscle myotube plasma membranes, as revealed by α-BTX staining (red), presumably due to neural agrin present in the culture medium. This suggests nerve-contact-independent nAChR clustering in our 3D experimental model.

### 2.6. 3D Reconstruction and Morphology Analysis

**Figure 9:**
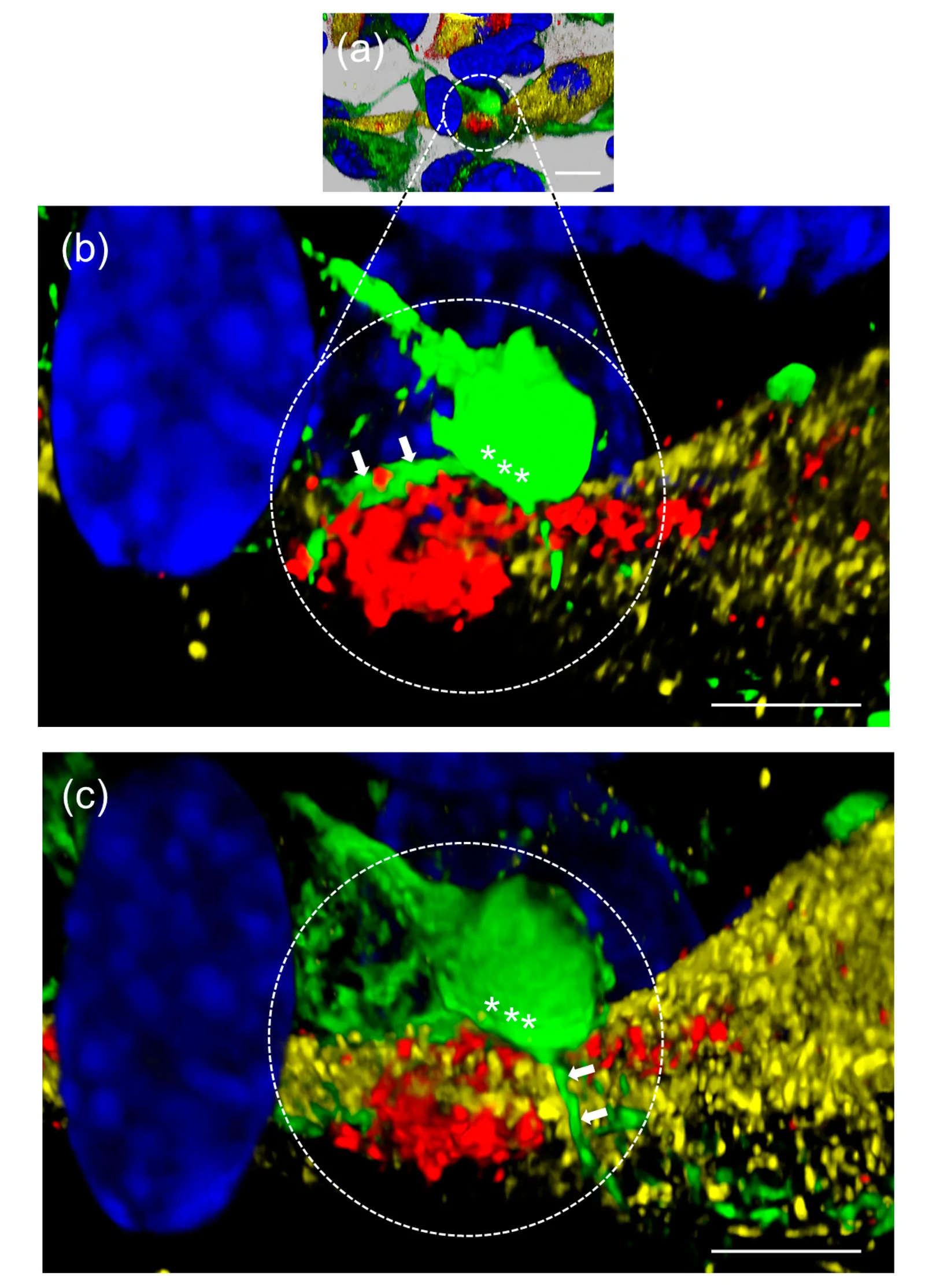

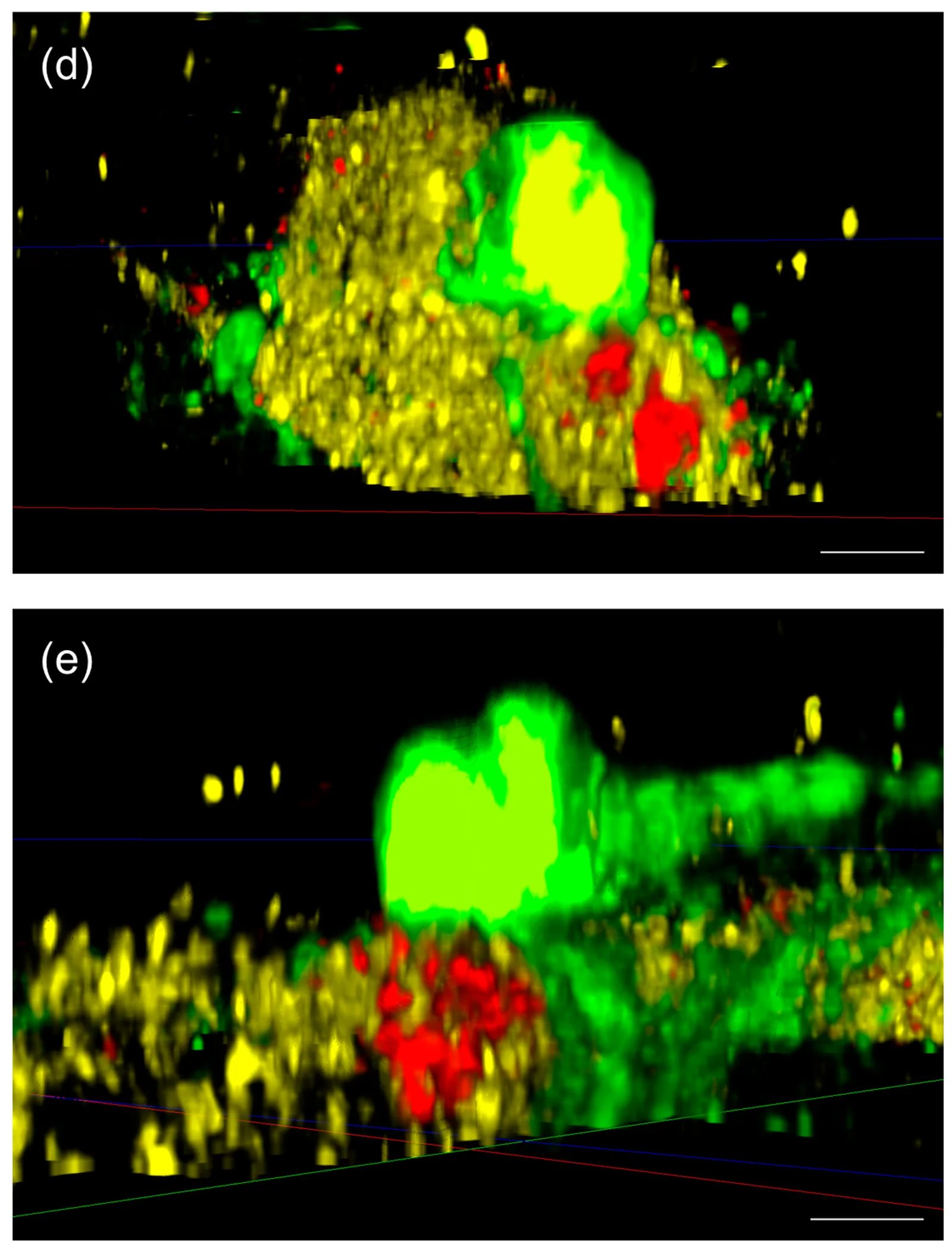
Nerve and muscle cell–cell contact morphology analysis by laser confocal microscopy. (**a**) An overview of a C2C12 myotube and NSC-34 motoneuron in 3D co-culture on a synthetic Alvetex scaffold. Immunofluorescence staining was performed for neurofilaments (green) and muscle desmin (yellow), and nAChRs were stained with α-BTX (red). Nuclei were counterstained with DAPI (blue). Magnification bar = 12 µm. (**b**) After deconvolution image analysis, zoom in on the area enclosed by the dashed circle in the upper panel. The close apposition of green and red staining is clearly visible (asterisks). The arrows indicate an area corresponding to the branches of the terminal bud. Magnification bar = 5 µm. (**c**) The same image as in panel (**b**), but with the contrast and brightness adjusted to reveal an additional nerve ending branch (arrows). Magnification bar = 5 µm. (**d**,**e**) Two x-z orthogonal reconstructions of the same image as in (**c**). Color lines outline the boundary of the NMJ region used for the analysis. Magnification bar = 2.5 µm.

Morphology data analysis was performed using the Leica LAS X work package in combination with a deconvolution algorithm (the STED Xtend algorithm) in order to further investigate the juxtaposition of adjacent cells. Figure 9 illustrates the results obtained by z-image stack 3D reconstruction representative of adjacent NSC-34 motoneurons and C2C12 myotubes observed on polystyrene synthetic scaffolds, indicating the formation of synapse-like structures. Deconvolution image analysis revealed NSC-34 motoneuron branching as in the NMJ in vivo, validating the reliability of our 3D NSC-34/C2C12 co-culture experimental model.

### 2.7. Western Blot Analysis

Total protein extracts from both C2C12 myotube monocultures and NSC-34/C2C12 co-cultures in hydrogel were investigated for desmin expression patterns. Desmin is a known early marker of skeletal muscle development and plays a key role in the stability of muscle sarcomeres and membrane architecture.

**Figure 10:**
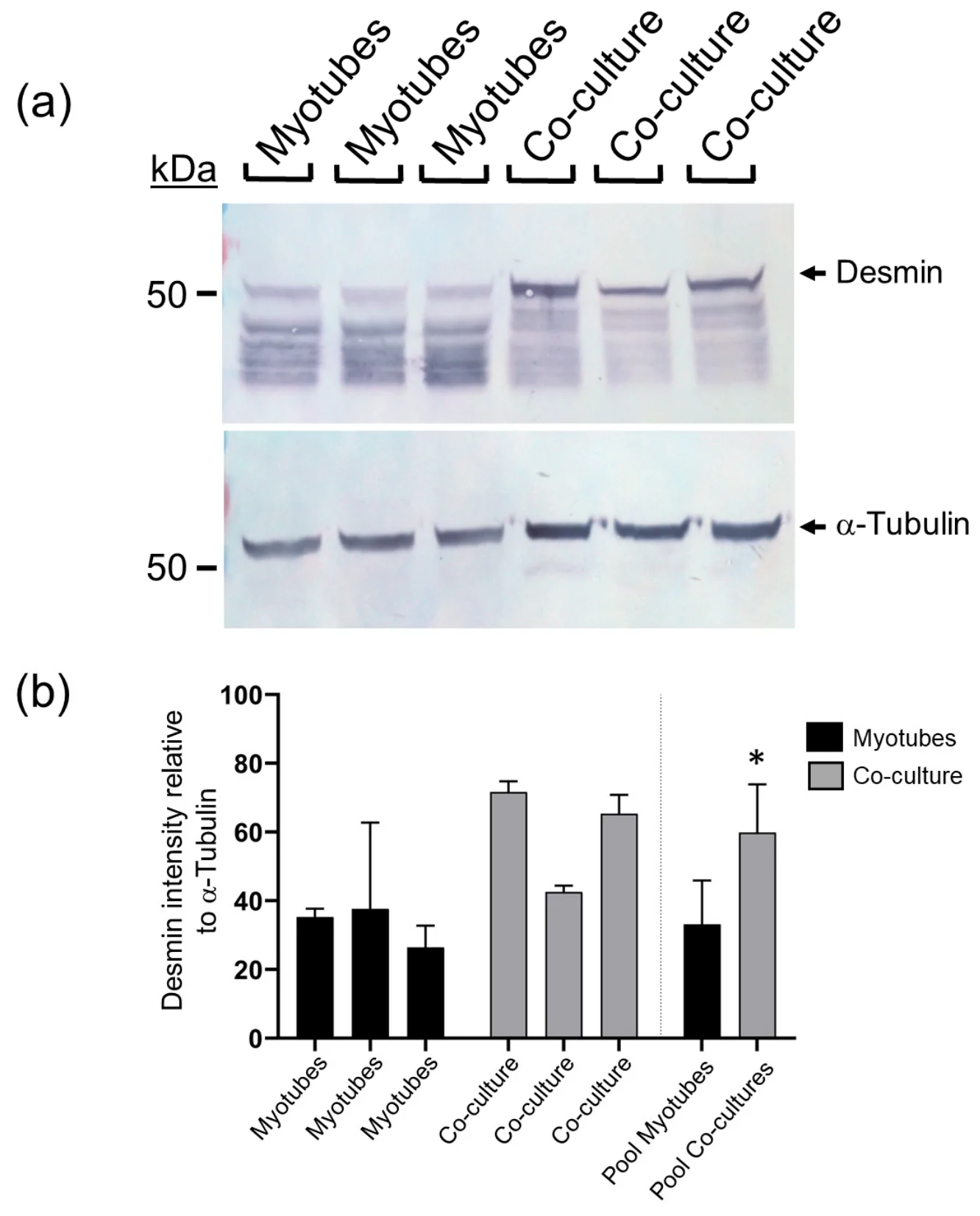
Desmin Western blot analysis of monocultured C2C12 myotubes (myotubes) and co-cultured myotubes with NSC-34 motoneurons (Co-culture). (**a**) The upper panel shows representative Western blots of three replicates of cell extracts from monocultured C2C12 myotubes (lanes 1–3) and co-cultured myotubes (lanes 4–6). A 53 kDa immunoreactive band, consistent with desmin’s relative mobility (upper band), is present in all lanes. (**a**) Lower panel: Western blot α-tubulin internal loading control. (**b**) Densitometric analysis of the 53 kDa desmin content. The results were normalized to α-tubulin and expressed as the mean ± SE of at least three independent experiments, each with three replicates. Asterisk represent significance of Pool Co-cultures vs. Pool Myotubes. *p* = 0.006.

Desmin content was analyzed using Western blot and a specific monoclonal anti-desmin antibody. As shown in Figure 10a, a desmin immunoreactive band consistent with the predicted MW of desmin of 53 kDa (Figure 10a: upper band, arrow), was present in all myotubes, whether monocultured or co-cultured. Densitometric analysis revealed significant differences between monocultures and co-cultures (Figure 10b). Notably, densitometric analysis showed an increase in desmin protein in co-cultured myotubes or myotubes grown in the presence of NSC-34 motoneurons compared to myotube monocultures. These results are consistent with increased myogenic activity in 3D co-cultured myotubes compared to myotube monocultures.

### 2.8. Quantitative PCR (qPCR) Gene Expression Analysis

**Figure 11:**
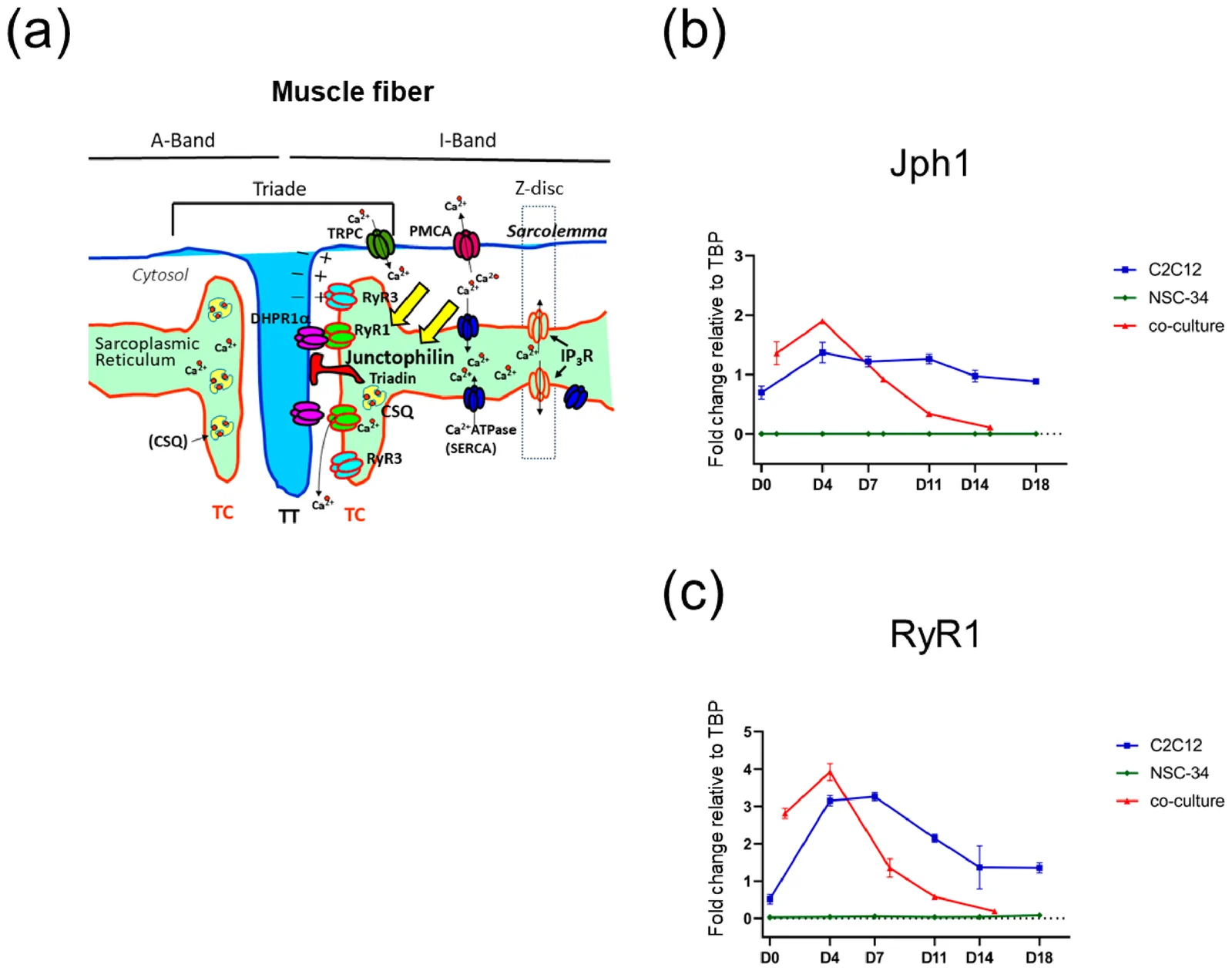
qPCR transcription analysis of RyR1, jph1, and SNAP25. (**a**) Diagram representing molecular players including Jph1 and RyR1 (yellow arrow) at the muscle triadic junction. (**b**) qPCR analysis of the triadic junction protein Jph1 in C2C12 myotubes 3D monoculture, NSC-34 motoneurons 3D monoculture and 3D NSC-34/C2C12 co-culture. (**c**) qPCR analysis of RyR1 in C2C12 myotubes 3D monoculture, NSC-34 motoneurons 3D monoculture and 3D NSC-34/C2C12 co-culture. Dotted line represents the x-axis. Results are the mean ± SE of at least three independent experiments with a duplicate for each experimental culture condition.

Levels of jph1, RyR1 and SNAP25 mRNA transcripts were measured in total RNA extracts from C2C12 and NSC-34 monocultures and NSC-34/C2C12 co-cultures at in vitro days 0, 4, 7, 11, 14, and 18 for monocultures and days 1, 4, 8, 11, and 15 for co-cultures. The results clearly showed that in the presence of NSC-34 motoneurons, myotubes exhibited a slight increase in transcription of both jph1, an important component of the triadic junction, and RyR1, the major intracellular calcium release channel regulating muscle contraction. The expression of both proteins in vitro reflects the increased organization and maturation of the triadic junction and excitation-contraction coupling machinery (Figure 11a–c).

**Figure 12:**
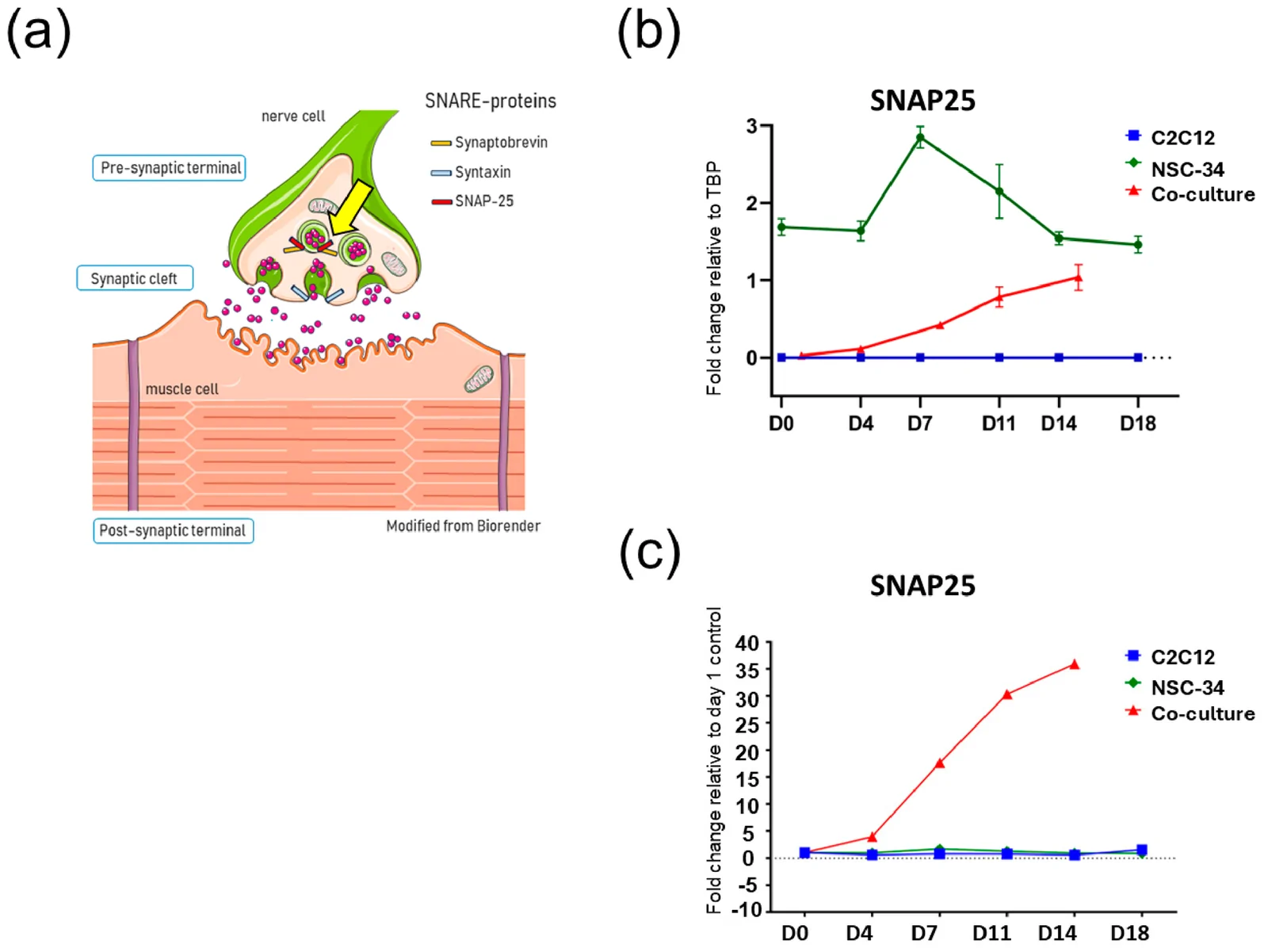
qPCR transcription analysis of SNAP-25. (**a**) Diagram representing the role of SNARE proteins at the presynaptic side (yellow arrow). (**b**) qPCR analysis of SNAP25 in C2C12 myotubes 3D monoculture, NSC-34 motoneurons 3D monoculture and 3D C2C12/NSC-34 co-culture. (**c**) qPCR analysis of the SNARE protein SNAP-25 in C2C12 myotubes 3D monoculture, NSC-34 motoneurons 3D monoculture and 3D C2C12/NSC-34 co-culture. Dotted line represents the x-axis. Values were normalized to Day-0 control.

Similarly, elevated transcription of SNAP25 (a SNARE family presynaptic vesicle key protein) was found in NSC-34 motoneurons and NSC-34/C2C12 co-cultures (Figure 12a–c). Figure 12c shows an approx. 20-fold increase in SNAP25 transcriptional activity in motoneurons from day 4 to day 14 co-cultures compared to monocultures, as assessed by qPCR. Data were normalized to in vitro day 0 control groups (C2C12 myotubes and NSC-34 motoneurons in monoculture). As expected, SNAP25 transcription was upregulated in co-cultures.

Thus, transcription regulation of key pre- and postsynaptic developmental proteins was consistent with NMJ maturation.

## 3. Discussion

Here we report on the comprehensive molecular and cellular characterization of an in vitro 3D C2C12/NSC-34 co-culture model grown in a biological hydrogel matrix. This model is a novel, standardized, and highly reliable tool for modeling the structure, development and functional adaptation of the NMJ in peripheral neuromuscular communication. The established co-culture model, embedded in an optimized, highly viscous biological hydrogel, provided reliable micro-morphometric evidence (using high-resolution differential interference contrast/laser confocal microscopy), as well as multiple molecular signatures of proper in vitro cell-to-cell contact assembly. This included pre- and postsynaptic protein biomarkers, such as SNAP-25/AChR, and specific triad junction proteins Jph-1/RyR1. The 3D cell model appears to recapitulate both initial stages of neuromuscular synaptogenesis (axonal outgrowth and target cell contact) and its subsequent consolidation under highly controlled in vitro conditions. This established 3D scaffold-based in vitro model could be used for various experimental applications in research laboratories and may also serve as a feasible, resilient and reproducible cell model for microgravity research in spaceflight.

Morphology analysis and cellular characterization in 3D co-cultures. Clusters or patterned nAChRs progressively increased on the C2C12 myotubes’ plasma membrane and were subsequently approached by NSC-34 motoneuron terminals, supporting the myocentric model [22,23,24] as documented by morphological, cytochemical and immunocytochemical analyses.

Moreover, live cell imaging showed an apparent increase in C2C12 myotube contractility in co-cultures above the spontaneous threshold (Salanova’s lab communication). However, an accurate quantification was not performed because this is an issue that will be addressed in a subsequent study. Furthermore, innervated C2C12 myotubes were characterized by increased expression of both desmin and fMyHC (both specific muscle differentiation markers). In addition, morphological analysis revealed in vitro differentiation of C2C12 myotubes displaying the typical cross-striated pattern or sarcomere-like structures, resulting from a developmentally driven increase in muscle-nerve cell–cell contact. Interestingly, high-resolution DIC images unequivocally showed that large NSC-34 motoneurons with elongated and branched axons (i.e., sprouting) were in close contact with several maturing C2C12 myotubes nearby. Similar organizations are typically known as “motor units” in normal skeletal muscle physiology.

In both types of 3D scaffolds, high-resolution morphological data analysis (DIC and laser confocal microscopy) revealed the presence of adjacent C2C12 and NSC-34 cells. Unfortunately, due to limitations imposed by the hydrogel matrix, electrophysiological data could not be obtained on the co-cultures. Thus, we might only speculate that in our 3D cultures, cell–cell contacts were also functional.

Additionally, quantitative RT-PCR analysis revealed that C2C12/NSC-34 co-cultured cells exhibited a 20-fold increase in SNAP25 mRNA transcription and slight transient increases in the triad junction-specific proteins Jph1 and RyR1. Given that only 10% to 20% of the cell co-culture is represented by motoneurons, and given their difference in terms of cell size with respect to myotubes, then the observed changes in mRNA transcription of SNAP25 might be very representative. Moreover, given the essential role of SNAP25 at presynaptic plasma membrane active zones, the increased transcriptional activity of this protein in co-cultured nerve cells fully correlated with the morphological, morphometric, and immunocytochemical results, suggesting that our in vitro 3D co-culture model is able to recapitulate proper structural and molecular assembly of NMJ-like contacts.

Based on the present results, it is reasonable to hypothesize that NEMUCO is a reliable preclinical platform for modeling the NMJ in vitro and studying muscle unloading, aging, and spaceflight-related adaptations. Furthermore, the potential translational relevance of NEMUCO in various muscular dystrophies such as Amyotrophic Lateral Sclerosis (ALS) and Duchenne, as well as in autoimmune diseases, such as Myasthenia Gravis (MG), is equally important.

2D and 3D NMJ modeling in vitro. Several studies have previously attempted to model the NMJ in vitro using 2D and 3D co-cultures with either primary or immortalized nerve and muscle cell lines, or alternatively, induced pluripotent stem cell (iPSC)-derived organoids, for both functional and regulatory studies. A large body of evidence supports the reliability of 2D and 3D in vitro models for studying neuromuscular diseases involving NMJ injury or dysfunction, including peripheral motoneuron disorders and neurodegenerative and neuroimmunological diseases [25,26,27,28,29,30]. However, while such in vitro systems employ sophisticated tissue engineering tools and cell networks closely resembling in vivo systems, especially for organoids, they often lack reproducibility and standardization, resulting in inconsistent data collection and interpretation. Interestingly, a parallel study using the same nerve (NSC-34) and muscle (C2C12) cell types in a transwell co-culture system and 2D migration co-culture demonstrated significant activation of myogenesis and synaptogenesis in co-cultured versus monocultured cells [31], corroborating the results reported in the present study. However, the transwell method is better characterized for studying chemotaxis in cells with invasive properties, such as tumor cells [32], rather than physical cell–cell contacts in co-culture [31]. Moreover, morphological/histochemical data provide evidence of α-BTX-labeled nAChR clusters with no co-staining of specific axonal markers. Therefore, there is no direct evidence of the presence of NMJ-like structures in the transwell co-culture system. Moreover, according to the “myocentric” model [22,33], the authors cannot fully exclude the hypothesis that this is the result of aneural prepatterned AChR clusters. Notably, Saini et al. [34] co-cultured human immortalized muscle cells with rat motoneurons, whereas Guo et al. [35] presented a model of human muscle cells co-cultured with motoneurons derived from human stem cells.

Although many previous studies have successfully modeled the NMJ in vitro in 2D and 3D, none have demonstrated it with such a high level of pre- and postsynaptic resolution and comprehensive characterization as presented here at structural, morphological, immunocytochemical, and molecular levels. Thus, we conclude that our experimental model represents a reliable and powerful tool for exploring in vitro molecular mechanisms of NMJ adaptation and plasticity under healthy culture conditions and in different pathophysiological settings, such as neurodegenerative and autoimmune diseases like myasthenia gravis [36]. Alternatively, it can be applied under increased or decreased oxidative stress conditions or altered gravity conditions such as simulated and/or real microgravity (spaceflight experiments aboard the International Space Station, ISS), which in some ways mimic muscle unloading in patients on Earth suffering from chronic immobilization.

3D scaffolds for in vitro NMJ research. As shown in our results, the demonstrated cell viability and development of NMJ-like structures in a closed and automated cell support system such as the CellBox hardware [37] on Earth indicate that the NEMUCO experimental model represents a powerful and reliable platform for studying NMJ development, maturation, and stability under different environmental culture conditions, including chronic exposure to microgravity. In this regard, the “NEMUCO-Biomission Spaceflight Experiment” is part of the European Space Agency (ESA) Research Program, launched to the ISS in spring 2026. However, spaceflight data are not yet available due to procedural constraints.

Skeletal muscle adaptations during chronic disuse in various muscle disease conditions are sensed at the NMJ [1,2,7]. The most relevant changes include decreased numbers of synaptic vesicles and neurotransmitter content [38,39], degeneration of axonal terminals, and axonal sprouting [40]. Adult NMJs undergo continuous remodeling under normal physiological conditions. Extended periods of increased or decreased neuromuscular activity more extensively stimulate remodeling, including continuous changing of overall NMJ microstructure and size.

Morphological alterations of the NMJ in response to reduced neuromuscular activity and the role of exercise in maintaining integrity have been previously extensively described [39,41]. Interestingly, in all models of disuse-induced muscle atrophy (bed rest, denervation, limb immobilization), the NMJ experiences disruptive structural adaptations similar to those observed during microgravity exposure. For instance, loss of motor units following denervation and muscle unloading results in a metabolic shift from slow/oxidative toward fast/glycolytic phenotypes [40,42,43], as well as mitochondrial alterations and increased oxidative stress [44,45,46].

Skeletal muscle not only contracts but also maintains different mechanisms of intra- and extra-synaptic myonuclear gene expression and translational regulation, thus supporting synaptic plasticity. However, the mechanisms translating neuronal electrical stimuli into skeletal muscle transcriptional and mechanical responses in different physiological and/or pathophysiological processes are complex and require highly coordinated signaling pathways and their cross-talk.

Therefore, the molecular mechanisms and underlying factors that negatively influence NMJ performance during acute and/or chronic muscle unloading in health or neurodegenerative diseases, leading to muscle atrophy, remain largely unexplored. For this purpose, NEMUCO provides a reliable preclinical platform for studying the molecular mechanisms that affect the shuttling of specific transcription factors and/or kinase/phosphatase to subsynaptic myonuclei.

Study Limitations. Due to its unique complexity and methodological limitations, studying the human NMJ at the cellular and molecular level in vivo is challenging, especially regarding the effects of pharmacological treatments and testing. Therefore, information about fundamental aspects of NMJ structural and ultrastructural adaptations, development, maturation, and maintenance in vivo has primarily come from animal experimental models [38,47,48,49,50,51]. In conclusion, the availability of a reliable nerve and muscle 3D co-culture model, although it can only be used for a limited period of time compared to iPSC, provides a unique and valuable experimental tool for further investigating and better understanding the cellular and molecular mechanisms of NMJ adaptation and plasticity. This model is a powerful alternative to animal models.

## 4. Materials and Methods

### 4.1. C2C12 Cell Culture

Murine C2C12 myoblasts (ATCC, CRL-1772) were plated in T75 flasks coated with 0.2% gelatin and maintained in growth medium (GM), Dulbecco’s modified Eagle’s medium containing 4.5 g/L glucose (DMEM; Gibco, Thermo Fisher Scientific, Inc., Waltham, MA, USA, 11960044)) with 10% heat inactivated fetal bovine serum (FBS; Sigma, St. Louis, MO, USA, F9665) and Penicillin-Streptomycin-Glutamine (100x) (Gibco, 10378016) at 37 °C in an atmosphere of 5% CO_2_. Cells should be subcultured prior to reaching confluency (subculture at 60–70% confluency). Cells were detached with Trypsin/EDTA (0.5%) (Gibco, 15400054). Cell cultures are never grown beyond 90% confluency, as this will result in their differentiation into myotubes. Cultures were used until passage 3.

To induce differentiation, C2C12 cells were grown to 80% confluence prior to media change for differentiation medium (DM) standard DMEM (Gibco, 11960044) containing B27 (50x) (Gibco, 17504001), murine IGF-1 (Peprotech, Rocky Hill, NJ, USA, 250-19), BDNF (Stem Cell Technologies, Vancouver, BC, Canada, 78005), GDNF (Stemcell Technologies, 78058), retinoic acid (BioGems, Westlake Village, CA, USA, 3027949), and Penicillin-Streptomycin-Glutamine (100X) (Gibco). Cultures were replenished with fresh medium every two days.

### 4.2. NSC-34 Cell Culture

The motoneuron cell line NSC-34 (tebu-bio, CLU140) was seeded in T75 flasks and cultivated in GM, Dulbecco’s modified Eagle’s medium (DMEM) containing 4.5 g/L glucose (Gibco) with 10% fetal bovine serum (FBS) (Sigma) and Penicillin-Streptomycin-Glutamine (100X) (Gibco) at 37 °C in an atmosphere of 5% CO_2_. Cultures were used between 2 and 5 passages. NSC-34 cells were only differentiated on a surface coated with Matrigel (Corning Inc., Corning, NY, USA, 354234) in a differentiation medium (NBN2) (Neurobasalmedium (Gibco, 21103049) containing N2 (Gibco, 17502048), murine IGF-1 (Peprotech), MEM non-essential amino acids (Gibco), retinoic acid (BioGems) and Penicillin-Streptomycin-Glutamine (100X) (Gibco)). Cultures were replenished with fresh medium every two days. When neurite elongation was observed in most NSC-34 cells under a phase-contrast microscope, they were incubated for 12–18 h with GM containing 1 µg/mL Mitomycin C (Stemcell Technologies, 73274). The NSC-34 cells were then rinsed twice with GM, and cultivated for two more days in differentiation medium, NSC-34 cells were detached with Accutase (Gibco, A1110501) prior to co-culture with differentiating C2C12 cells.

### 4.3. 3D Co-Culture

C2C12 cells were seeded on a synthetic polystyrene 3D scaffold (Reprocell, www.reprocell.com) coated with Laminin (Gibco, 23017015) or Matrigel (Corning) and differentiation was initiated after 2 days of proliferation. NSC-34 cells were added after differentiation to myotubes in a 3 to 1 ratio. We tested different media for the co-culture to find the optimal environment for both cell types and finally used a modification of the co-culture medium from [52]. In addition, we added Penicillin-Streptomycin-Glutamine (100X) (Gibco) and Agrin (R&D Systems, Inc. Minneapolis, MN, USA, 550-AG). Cultures were replenished with fresh medium every two days.

Subsequently, we seeded C2C12 in a biological scaffold, a hydrogel based on Collagen type I 36% (Merck Millipore, Darmstadt, Germany, 08115) and Matrigel 14% (Corning). C2C12 were proliferated in GM until 70% confluency was reached and differentiated in DM for one or two days. NSC-34 (two days after Mitomycin C treatment) were then seeded on top of the hydrogel in a 1:9 ratio. Medium was then changed to co-culture medium. Cells were grown on hydrogel for several days and analyzed at different time points (4D co-culture).

### 4.4. Immunohistochemistry and Immunocytochemistry

#### 4.4.1. Tissue Cryosection Preparation

The animal tissue used in this study was obtained from a previously conducted experiment [1] and only samples from the control group were used. Directly after the rats were euthanized, the SOL muscle was dissected and snap-frozen in liquid nitrogen and kept at −80 °C until cryo-sectioning. 10 μm thick sections were collected from the middle portion of the muscle belly using a cryostat (Leica Kryostat Frigocut 2800E, Reichert-Jung, Nußloch, Germany). The cryosections were processed according to the immunohistochemical protocol procedure previously reported by Salanova et al. [1].

#### 4.4.2. Immunohistochemistry Analysis

Tissue cryosection fixation was carried out in 4% phosphate-buffered formaldehyde (Histofix, Carl Roth, Karlsruhe, Germany) for 10 min at 4 °C, then washed in PBS. To visualize the presynaptic input, anti-neurofilament antibodies (Millipore, mAb5266, 1:200) were used. Primary antibodies were visualized using Alexa Fluor 488-conjugated secondary antibodies (Molecular Probes, A11029, 1:3000, Eugene, OR, USA). The α-bungarotoxin (α-BTX) conjugated with Alexa Fluor 594 (Invitrogen, B13423, 1:1000, Waltham, MA, USA) was used, as it specifically binds to acetylcholine receptors (AChRs) located at the motor endplate of the NMJ. Nuclei were counterstained with DAPI (Invitrogen, D1306, 1:1000, Waltham, MA, USA).

#### 4.4.3. Immunocytochemistry Analysis

Cultured cells were fixed with ice-cold Roti^®^Histofix (4% formaldehyde) (Carl Roth GmbH + Co. KG, Karlsruhe, Germany, P087.1) at different time points and investigated by immunohistochemistry using different muscle- and nerve-specific differentiating markers. Prior to staining, the cells were permeabilized and blocked with a blocking buffer containing 0.3% Triton^®^ X-100 (Sigma, 23,472-9), 2% normal goat serum (Invitrogen, 50-197Z) and 2% BSA in 1x TBS (pH 7.4) for 1 h to inhibit nonspecific protein interaction. The samples were then stained using monoclonal antibodies specific for desmin (1:50; Novocastra Laboratories, Newcastle, UK, NCL-L-DES-DERII), skeletal Myosin (fast) (1:1000; Sigma, M4276) and Neurofilament (1:200; EMD Millipore Corp., Temecula, CA, USA, MAB5266) overnight at 4 °C to visualize the junction of developing skeletal muscle fibers with the axons of the motoneurons. The next day, the samples were rinsed in TBS thrice for 2–3 min each to remove any unbound primary antibodies. Subsequently, the slides were incubated in either Alexa 488-conjugated goat anti-mouse IgG (Invitrogen, B13422), Alexa 555-conjugated goat anti-rabbit IgG (Invitrogen, B35451) or Alexa 635-conjugated goat anti-rabbit IgG (Invitrogen, A31575) at a 1:1000 dilution at room temperature for 1 h in the dark. In case of multiple first antibodies, there was an additional blocking step with the Mouse Ig Blocking Reagent M.O.M. (Vector Laboratories, Burlingame, CA, USA, MKB-2213) for 2 h in between. At last, the samples were stained with either Alpha-Bungarotoxin Conjugates Alexa Flour^®^ 488 (1:1000; Invitrogen, B13422) or Alpha-Bungarotoxin Conjugates Alexa Flour^®^ 555 (1:1000; Invitrogen, B35451) for 2 h in the dark. The rinsing step was performed as before and samples were stained for nuclei using the mounting medium with DAPI (Vector Laboratories, H-1400) and covered with cover slips. Primary antibodies were omitted in control experiments. Samples incubated with only secondary antibodies were used as controls. All the antibodies were diluted in 2% BSA in TBS prior to use.

### 4.5. Image Acquisition and Analysis

Confocal images were acquired at 63x (Objective HC PL APO CS2 63x/1.30 GLYC). Digitized z-stack images were acquired at high resolution (pixel size 90.19 × 90.19 nm) using the Leica TCS SP8 confocal microscope and further processed with the Leica Application Suite X 3.5.2.18963 (LAS X) software. Z-stack images were generated using optical sectioning with a pinhole size of 1 AU. The 2D images are maximal projections from the scanned optical sections.

The 3D images and videos were captured in the 3D software to better define the viewing angle for better 3D spatial perception of the adjacent nerve and muscle cells. For Figure 9 only, the image deconvolution was obtained by using the STED Xtend algorithm (TauSTED Xtend: gentle multicolor imaging at nanoscale resolution).

### 4.6. Western Blot Analysis

Total cell extracts were obtained using the RIPA buffer as Lysis Buffer containing (1% Triton X-100, 150 mM NaCl, 50 mM TRIS-HCL pH 7.4, 1% (*w*/*v*) NP-40, 0.1% (*w*/*v*) SDS, 1% (*w*/*v*) Na-Deoxycholate plus protease inhibitors (cOmplete Tablet EASYpack, Roche, LOT 41353800) 10 µL/mg tissue ratio.

Cell sample extracts were mechanically dissolved in the lysis buffer using a tight glass Dounce homogenizer (with 20 pestles) on ice.

After 20 min incubation in the Lysis Buffer on ice, samples were centrifuged with 14,000× *g* for 20 min at 4 °C. The supernatant fraction was carefully collected and stored at −80 °C until used.

Total protein concentration was estimated using the BCA Protein Assay Kit [53] and measured with NanoDrop One (Thermo Fisher Scientific, Waltham, MA, USA) and adjusted to the same protein concentration (1 mg/mL) with Laemmli sample buffer (0.5 M TRIS, 50% (*v*/*v*) Glycerol, 4% (*w*/*v*) SDS and 0.02% (*w*/*v*) Bromphenolblue) in the presence of reducing agents (700 mM DDT, 10% ß-Mercaptoethanol). Reducing and denaturing conditions included an additional heating step to 95 °C for 10 min.

The same amount of protein samples was loaded onto a 10% Bis-Tris gel and SDS-PAGE was performed. The gel was blotted on a PVDF membrane (0.45 µm) at 100 V for 60 min at 15 °C. Preincubation with a blocking solution containing 2% (*w*/*v*) skim milk powder (SERVA, 425590.01, LOT 200120). Incubation with primary antibodies was done overnight at room temperature. Specific mouse anti-desmin IgG1 antibodies were used from (MONOSAN Xtra, Clone DE-R-11, Cat. No. MONX10657) to detect the desmin protein. α-tubulin was used as an internal loading control (Sigma, T6199; https://www.sigmaaldrich.com, accessed on 9 April 2026).

Primary antibodies were visualized by using rabbit anti-mouse alkaline phosphatase-conjugated secondary antibodies (DAKO D0306, 1:500, 2 h, at RT) followed by incubation with an alkaline phosphatase substrate detection system (NBT/BCIP, #34042, Thermo Scientific, USA). The membranes were scanned with the Azure400 Fluorescent Imager (Azure Biosystems, Dublin, CA, USA) and analyzed with ImageJ 1.54 Software [54]. The image has been subjected first to channel splitting, followed by a background subtraction with a rolling ball radius of 50 pixels and a selected sliding paraboloid. The ROIs with cleared signal were set by the Threshold function with a dark background, applied and after the Analyze Particles function, the ROIs were added to the ROI Manager. The selected ROIs have been analyzed on the raw image using the Measure function, focusing on the area of signal, mean gray scale value, max and min gray scale value and integrated density as the total fluorescence signal within the selected ROIs.

### 4.7. qPCR Analysis

Total RNA from the cells in hydrogel was extracted using Trizol^®^ (Invitrogen; Thermo Fisher Scientific, Inc., 15596026) and the PureLink RNA Mini Kit (Invitrogen, 12183025) according to the manufacturer’s protocol. All steps after RNA extraction leading to the RNA sequencing were handled by the Charité Berlin Institute of Health (BIH) Core Facility Genomics.

### 4.8. RNA Extraction and Quantitative PCR (qPCR)

Total RNA was obtained from cell cultures (2 different time-course samples for each cell line and for co-culture were used) using the TRIzol^®^ (Thermo Fisher Scientific, USA) extraction method, according to the manufacturer’s instructions. 1 mg of RNA was converted to cDNA using random hexamers and SuperScript^®^ VILO™ (Thermo Fisher Scientific, USA) following the manufacturer’s instructions. Specific primers for SNAP25, Jph1 and RyR1 were designed using Primer3, Release 2.6.0 software (https://primer3.org/). Thermodynamic specificity was determined using BLAST sequence alignment (https://blast.ncbi.nlm.nih.gov/, accessed on 6 March 2026) and vector NTI^®^ software (Vector NTI Advance v11.5.2; https://vector-nti.software.informer.com/, accessed on 6 March 2026).

Primer sequences were as follows:
m_SNAP25_fw5′-CGCAATGAGCTGGAGGAGAT-3′m_SNAP25_rv5′-TCGCCTTGCTCATCCAACAT-3′m_Jph1_fw5′-GCAAGCAATGATACCTGCCC-3′m_Jph1_rv5′-AAGGCTATGCTTACCTGTCCTAA-3′m_RyR1_fw5′-GGCAGATGAGAAGGCAGAAG-3′m_RyR1_rv5′-TCCACCAGAGTTGTCTGTGC-3′

qPCR was performed in a CFX Connect^®^ Real-time System (Bio-Rad, Hercules, CA, USA) using SYBR Green chemistry. All samples were run in triplicate and simultaneously with RNA-negative controls. Cyclophilin A (PPIA), Hypoxanthine Phosphoribosyl transferase 1 (HPRT1) and TATA-Box Binding Protein (TBP) were tested as candidate reference genes, being the latter being the most stable for normalizing Ct values. Primer sequences were published elsewhere [55]. Normalization was performed by the ΔCt method.

### 4.9. Statistical Analysis

The Linear Mixed Effects Model was used for statistical analysis. Data analysis was performed using GraphPad Prism 10.5.0 software. Results were considered statistically significant at a predefined level of *p* < 0.05 confidence. All graphs were created with GraphPad Prism5 software.

## 5. Conclusions

In light of the growing demand for in vitro experimental models and tissue engineering methodologies, particularly for physiological, pathophysiological and pharmacological studies, we have developed a robust and highly standardized tool (3D NErve and MUscle CO-culture, NEMUCO) for studying the structural and molecular evolution over time (the 4th dimension, 4D co-culture). This model provides deeper insights into the major characteristics of NMJ synaptogenesis, from microarchitecture to underlying mechanisms, as reflected by defined molecular expression profiles. These profiles are replicated under well-defined 3D in vitro culture conditions. In vitro modeling of the NMJ represents a useful tool for studying the cellular, subcellular and molecular events that lead to subcellular organization of key molecular players in pre- and postsynaptic NMJ microdomains. It is applicable in various experimental settings, from fundamental to clinical research laboratories.

In addition to cell models grown on specialized biological chips [26,56], the standardized 3D neuron-muscle co-cultures reported here, derived from well-characterized single cell lines, provide a reliable research platform for studying the temporal and spatial assembly of molecules that regulate synaptogenesis, NMJ stability, adaptation and plasticity. The NEMUCO model can also be used to test therapeutic pharmacological approaches or treatments capable of restoring impaired NMJ transmission in various neurodegenerative clinical settings, including autoimmune neuromuscular diseases such as myasthenia gravis, as well as conditions involving altered muscle activity in microgravity and/or during aging.

## Data Availability

The original contributions presented in this study are included in the article. Further inquiries can be directed to the corresponding author.

## References

[1] Salanova M., Bortoloso E., Schiffl G., Gutsmann M., Belavy D.L., Felsenberg D., Furlan S., Volpe P., Blottner D. (2011). Expression and regulation of Homer in human skeletal muscle during neuromuscular junction adaptation to disuse and exercise. FASEB J..

[2] Iyer S.R., Shah S.B., Lovering R.M. (2021). The Neuromuscular Junction: Roles in Aging and Neuromuscular Disease. Int. J. Mol. Sci..

[3] Huijbers M.G., Marx A., Plomp J.J., Le Panse R., Phillips W.D. (2022). Advances in the understanding of disease mechanisms of autoimmune neuromuscular junction disorders. Lancet Neurol..

[4] Murgia M., Rittweger J., Reggiani C., Bottinelli R., Mann M., Schiaffino S., Narici M.V. (2024). Spaceflight on the ISS changed the skeletal muscle proteome of two astronauts. npj Microgravity.

[5] Blottner D., Moriggi M., Trautmann G., Hastermann M., Capitanio D., Torretta E., Block K., Rittweger J., Limper U., Gelfi C. (2023). Space Omics and Tissue Response in Astronaut Skeletal Muscle after Short and Long Duration Missions. Int. J. Mol. Sci..

[6] Bao Z., Cui C., Chow S.K., Qin L., Wong R.M.Y., Cheung W.H. (2020). AChRs Degeneration at NMJ in Aging-Associated Sarcopenia-A Systematic Review. Front. Aging Neurosci..

[7] Blottner D., Trautmann G., Furlan S., Gambara G., Block K., Gutsmann M., Sun L.W., Worley P.F., Gorza L., Scano M. (2021). Reciprocal Homer1a and Homer2 Isoform Expression Is a Key Mechanism for Muscle Soleus Atrophy in Spaceflown Mice. Int. J. Mol. Sci..

[8] Moss K.R., Darvishi F.B., Badawi Y., Fish L.A., Funke J.R., Pedersen T.H., Robitaille R., Arnold W.D., Burgess R.W., Meriney S.D. (2025). The Neuromuscular Junction: A Shared Vulnerability in Aging and Disease. J. Neurosci..

[9] Sanes J.R., Lichtman J.W. (1999). Development of the vertebrate neuromuscular junction. Annu. Rev. Neurosci..

[10] Davis L.A., Fogarty M.J., Brown A., Sieck G.C. (2022). Structure and Function of the Mammalian Neuromuscular Junction. Compr. Physiol..

[11] Jimsheleishvili S., Marwaha K., Sherman A.L. (2026). Physiology, Neuromuscular Transmission.

[12] Hyman S.E. (2005). Neurotransmitters. Curr. Biol..

[13] Mukund K., Subramaniam S. (2020). Skeletal muscle: A review of molecular structure and function, in health and disease. WIREs Biol. Med..

[14] Mis K., Grubic Z., Lorenzon P., Sciancalepore M., Mars T., Pirkmajer S. (2017). In Vitro Innervation as an Experimental Model to Study the Expression and Functions of Acetylcholinesterase and Agrin in Human Skeletal Muscle. Molecules.

[15] Andersen J., Revah O., Miura Y., Thom N., Amin N.D., Kelley K.W., Singh M., Chen X., Thete M.V., Walczak E.M. (2020). Generation of Functional Human 3D Cortico-Motor Assembloids. Cell.

[16] Barbeau S., Tahraoui-Bories J., Legay C., Martinat C. (2020). Building neuromuscular junctions in vitro. Development.

[17] Zhang K., Bai L., Xu W., Shen C. (2022). Human neuromuscular junction three-dimensional organoid models and the insight in motor disorders. J. Mol. Cell Biol..

[18] Yaffe D., Saxel O. (1977). Serial passaging and differentiation of myogenic cells isolated from dystrophic mouse muscle. Nature.

[19] Nango H., Kosuge Y., Sato M., Shibukawa Y., Aono Y., Saigusa T., Ito Y., Ishige K. (2020). Highly Efficient Conversion of Motor Neuron-Like NSC-34 Cells into Functional Motor Neurons by Prostaglandin E_2_. Cells.

[20] Knight E., Murray B., Carnachan R., Przyborski S. (2011). Alvetex^®^: Polystyrene scaffold technology for routine three dimensional cell culture. 3D Cell Culture.

[21] Zhang Y., Xu Y., Gao J. (2023). The engineering and application of extracellular matrix hydrogels: A review. Biomater. Sci..

[22] Lin S., Landmann L., Ruegg M.A., Brenner H.R. (2008). The role of nerve- versus muscle-derived factors in mammalian neuromuscular junction formation. J. Neurosci..

[23] DeChiara T.M., Bowen D.C., Valenzuela D.M., Simmons M.V., Poueymirou W.T., Thomas S., Kinetz E., Compton D.L., Rojas E., Park J.S. (1996). The receptor tyrosine kinase MuSK is required for neuromuscular junction formation in vivo. Cell.

[24] Yang X., Arber S., William C., Li L., Tanabe Y., Jessell T.M., Birchmeier C., Burden S.J. (2001). Patterning of muscle acetylcholine receptor gene expression in the absence of motor innervation. Neuron.

[25] Kim H., Kim G.S., Hyun S.H., Kim E. (2023). Advancements in 2D and 3D In Vitro Models for Studying Neuromuscular Diseases. Int. J. Mol. Sci..

[26] Leng Y., Li X., Zheng F., Liu H., Wang C., Wang X., Liao Y., Liu J., Meng K., Yu J. (2023). Advances in In Vitro Models of Neuromuscular Junction: Focusing on Organ-on-a-Chip, Organoids, and Biohybrid Robotics. Adv. Mater..

[27] Nebol A., Gouti M. (2024). A new era in neuromuscular junction research: Current advances in self-organized and assembled in vitro models. Curr. Opin. Genet. Dev..

[28] Luttrell S.M., Smith A.S.T., Mack D.L. (2021). Creating stem cell-derived neuromuscular junctions in vitro. Muscle Nerve.

[29] Qaisar R. (2023). Targeting neuromuscular junction to treat neuromuscular disorders. Life Sci..

[30] Lotti F., Przedborski S. (2022). Motoneuron Diseases. Vertebrate Motoneurons.

[31] Redden J.T., Kothe S., Cohen D.J., Schwartz Z., McClure M.J. (2025). Trophic Factors in Muscle-Nerve Cross-Talk Signaling Augment Muscle Fiber and Motor Endplate Development. J. Cell. Physiol..

[32] Justus C.R., Marie M.A., Sanderlin E.J., Yang L.V. (2023). Transwell In Vitro Cell Migration and Invasion Assays. Cell Viability Assays.

[33] Liu Y., Padgett D., Takahashi M., Li H., Sayeed A., Teichert R.W., Olivera B.M., McArdle J.J., Green W.N., Lin W. (2008). Essential roles of the acetylcholine receptor γ-subunit in neuromuscular synaptic patterning. Development.

[34] Saini J., Faroni A., Abd Al Samid M., Reid A.J., Lightfoot A.P., Mamchaoui K., Mouly V., Butler-Browne G., McPhee J.S., Degens H. (2019). Simplified in vitro engineering of neuromuscular junctions between rat embryonic motoneurons and immortalized human skeletal muscle cells. Stem Cells Cloning Adv. Appl..

[35] Guo X., Gonzalez M., Stancescu M., Vandenburgh H.H., Hickman J.J. (2011). Neuromuscular junction formation between human stem cell-derived motoneurons and human skeletal muscle in a defined system. Biomaterials.

[36] Schubert M., Pelz A., Trautmann G., Block K., Furlan S., Gutsmann M., Kohler S., Volpe P., Blottner D., Meisel A. (2022). Opposite Regulation of Homer Signal at the NMJ Postsynaptic Micro Domain between Slow- and Fast-Twitch Muscles in an Experimentally Induced Autoimmune Myasthenia Gravis (EAMG) Mouse Model. Int. J. Mol. Sci..

[37] Melnik D., Kruger M., Schulz H., Kopp S., Wehland M., Bauer J., Baselet B., Vermeesen R., Baatout S., Corydon T.J. (2021). The CellBox-2 Mission to the International Space Station: Thyroid Cancer Cells in Space. Int. J. Mol. Sci..

[38] Baranski S., Marciniak M. (1990). Morphometric ultrastructural analysis of muscle fibers and neuromuscular junction in rats kept under conditions of weightlessness for 21 days. Mater. Med. Pol..

[39] Deschenes M.R., Wilson M.H., Kraemer W.J. (2005). Neuromuscular adaptations to spaceflight are specific to postural muscles. Muscle Nerve.

[40] Pozdniakov O.M., Babakova L.L., Demorzhi M.S., Il’ina-Kakueva E.I. (1990). Changes in the ultrastructure of striated muscles and neuromuscular synapses of rats under the effects of a 13-day space flight. Kosm. Biol. Aviakosm. Med..

[41] Nishimune H., Stanford J.A., Mori Y. (2014). Role of exercise in maintaining the integrity of the neuromuscular junction. Muscle Nerve.

[42] Edgerton V.R., Roy R.R. (1994). Chapter 2 Neuromuscular adaptation to actual and simulated weightlessness. Advances in Space Biology and Medicine.

[43] Roy R.R., Baldwin K.M., Edgerton V.R. (1996). Response of the neuromuscular unit to spaceflight: What has been learned from the rat model. Exerc. Sport Sci. Rev..

[44] Salanova M., Gambara G., Moriggi M., Vasso M., Ungethuem U., Belavy D.L., Felsenberg D., Cerretelli P., Gelfi C., Blottner D. (2015). Vibration mechanosignals superimposed to resistive exercise result in baseline skeletal muscle transcriptome profiles following chronic disuse in bed rest. Sci. Rep..

[45] Antonutto G., Capelli C., Girardis M., Zamparo P., di Prampero P.E. (1999). Effects of microgravity on maximal power of lower limbs during very short efforts in humans. J. Appl. Physiol..

[46] Edgerton V.R., Zhou M.Y., Ohira Y., Klitgaard H., Jiang B., Bell G., Harris B., Saltin B., Gollnick P.D., Roy R.R. (1995). Human fiber size and enzymatic properties after 5 and 11 days of spaceflight. J. Appl. Physiol..

[47] Baranski S., Baranska W., Marciniak M., Ilyina-Kakueva E.I. (1979). Ultrasonic investigations of the soleus muscle after space flight on the Biosputnik 936. Aviat. Space Environ. Med..

[48] D’Amelio F., Daunton N.G. (1992). Effects of spaceflight in the adductor longus muscle of rats flown in the Soviet Biosatellite COSMOS 2044. A study employing neural cell adhesion molecule (N-CAM) immunocytochemistry and conventional morphological techniques (light and electron microscopy). J. Neuropathol. Exp. Neurol..

[49] Riley D.A., Ilyina-Kakueva E.I., Ellis S., Bain J.L., Slocum G.R., Sedlak F.R. (1990). Skeletal muscle fiber, nerve, and blood vessel breakdown in space-flown rats. FASEB J..

[50] Deschenes M.R., Britt A.A., Gomes R.R., Booth F.W., Gordon S.E. (2001). Recovery of neuromuscular junction morphology following 16 days of spaceflight. Synapse.

[51] Wilson M.H., Deschenes M.R. (2005). The neuromuscular junction: Anatomical features and adaptations to various forms of increased, or decreased neuromuscular activity. Int. J. Neurosci..

[52] Guo X., Das M., Rumsey J., Gonzalez M., Stancescu M., Hickman J. (2010). Neuromuscular junction formation between human stem-cell-derived motoneurons and rat skeletal muscle in a defined system. Tissue Eng. Part C Methods.

[53] Smith P.K., Krohn R.I., Hermanson G.T., Mallia A.K., Gartner F.H., Provenzano M.D., Fujimoto E.K., Goeke N.M., Olson B.J., Klenk D.C. (1985). Measurement of protein using bicinchoninic acid. Anal. Biochem..

[54] Schneider C.A., Rasband W.S., Eliceiri K.W. (2012). NIH Image to ImageJ: 25 years of image analysis. Nat. Methods.

[55] Gambara G., Salanova M., Ciciliot S., Furlan S., Gutsmann M., Schiffl G., Ungethuem U., Volpe P., Gunga H.C., Blottner D. (2017). Microgravity-Induced Transcriptome Adaptation in Mouse Paraspinal *Longissimus dorsi* Muscle Highlights Insulin Resistance-Linked Genes. Front. Physiol..

[56] Alavi-Moghadam S., Kokabi-Hamidpour S., Rezaei-Tavirani M., Larijani B., Arjmand R., Rahim F., Rezazadeh-Mafi A., Adibi H., Arjmand B. (2024). Neuromuscular Junction-on-a-Chip for Amyotrophic Lateral Sclerosis Modeling. Stem Cells and Lineage Commitment.

[57] Block K., Ziraldo G., Trautmann G., Furlan S., Singh A., Grosser S., Gutsmann M., Lorenzon P., Volpe P., Vida I. (2026). NEMUCO: The In Vitro 4D NMJ as an Innovative Powerful Tool to Study Nerve and Muscle Cell-Cell Communication. Preprints.org.

